# Cessation of Smoking Trial in the Emergency Department (COSTED): a multicentre randomised controlled trial

**DOI:** 10.1136/emermed-2023-213824

**Published:** 2024-03-26

**Authors:** Ian Pope, Lucy V Clark, Allan Clark, Emma Ward, Pippa Belderson, Susan Stirling, Steve Parrott, Jinshuo Li, Tim Coats, Linda Bauld, Richard Holland, Sarah Gentry, Sanjay Agrawal, Benjamin Michael Bloom, Adrian A Boyle, Alasdair J Gray, M Geraint Morris, Jonathan Livingstone-Banks, Caitlin Notley

**Affiliations:** 1 Norwich Medical School, University of East Anglia Norwich Medical School, Norwich, UK; 2 Department of Health Sciences, University of York, York, UK; 3 Department of Cardiovascular Sciences, University of Leicester, Leicester, UK; 4 Usher Institute, The University of Edinburgh, Edinburgh, UK; 5 University of Exeter, Exeter, UK; 6 Leicester Royal Infirmary, Leicester, UK; 7 Emergency Department, Barts Health NHS Trust, London, UK; 8 Emergency Department, Addenbrooke's Hospital, Cambridge, UK; 9 Emergency Department, Royal Infirmary of Edinburgh, Edinburgh, Edinburgh, UK; 10 Emergency Department, Homerton University Hospital NHS Foundation Trust, London, UK; 11 Nuffield Department of Primary Care Health Sciences, University of Oxford, Oxford, UK

**Keywords:** emergency departments, substance-related disorders, Clinical Trial

## Abstract

**Background:**

Supporting people to quit smoking is one of the most powerful interventions to improve health. The Emergency Department (ED) represents a potentially valuable opportunity to deliver a smoking cessation intervention if it is sufficiently resourced. The objective of this trial was to determine whether an opportunistic ED-based smoking cessation intervention can help people to quit smoking.

**Methods:**

In this multicentre, parallel-group, randomised controlled superiority trial conducted between January and August 2022, adults who smoked daily and attended one of six UK EDs were randomised to intervention (brief advice, e-cigarette starter kit and referral to stop smoking services) or control (written information on stop smoking services). The primary outcome was biochemically validated abstinence at 6 months.

**Results:**

An intention-to-treat analysis included 972 of 1443 people screened for inclusion (484 in the intervention group, 488 in the control group). Of 975 participants randomised, 3 were subsequently excluded, 17 withdrew and 287 were lost to follow-up. The 6-month biochemically-verified abstinence rate was 7.2% in the intervention group and 4.1% in the control group (relative risk 1.76; 95% CI 1.03 to 3.01; p=0.038). Self-reported 7-day abstinence at 6 months was 23.3% in the intervention group and 12.9% in the control group (relative risk 1.80; 95% CI 1.36 to 2.38; p<0.001). No serious adverse events related to taking part in the trial were reported.

**Conclusions:**

An opportunistic smoking cessation intervention comprising brief advice, an e-cigarette starter kit and referral to stop smoking services is effective for sustained smoking abstinence with few reported adverse events.

**Trial registration number:**

NCT04854616.

**WHAT IS ALREADY KNOWN ON THIS TOPIC**

- Emergency Department (ED)-based smoking cessation interventions have shown promise but there is uncertainty about the best intervention components and longer term outcomes.
- E-cigarettes have been shown to be one of the most effective smoking cessation tools but have never been tested in the ED environment.

**WHAT THIS STUDY ADDS**

- In this trial significantly more people receiving a smoking cessation intervention in the ED achieved long-term smoking abstinence compared with those receiving usual care. Findings were limited by relatively low biochemical validation rates and slightly differential rates of follow-up.
- This trial contributes to the existing evidence that ED-based interventions are effective and is the first trial to test e-cigarettes in the ED setting.

**HOW THIS STUDY MIGHT AFFECT RESEARCH, PRACTICE OR POLICY**

- Policy makers should consider the ED as a location to deliver smoking cessation interventions as long as appropriate funding is available for dedicated staff.
- This study shows that it is possible to recruit efficiently and to deliver a brief opportunistic intervention to support sustained tobacco smoking abstinence in the ED setting.

## Introduction

Tobacco kills more than 8 million people each year worldwide.^1^ In the UK 6.4 million people continue to smoke, with those in ‘routine and manual’ occupations having a smoking rate of 22.8% compared with 8.3% for those in ‘managerial and professional’ occupations.^2^ Treating tobacco addiction is a powerful tool to combat premature death, address health inequalities and to reduce healthcare utilisation.^3 4^ Emergency Departments (EDs) see large numbers of people, and those who attend the ED are more likely to smoke^5^ and suffer complex health inequalities.^6^

Smoking cessation interventions embedded in EDs have shown promise; however, there is uncertainty about the long-term impact and optimal intervention components.^7^ Previous studies in ED settings have evaluated behavioural support alone, or behavioural support combined with an offer of nicotine replacement therapy (NRT).^7^ Evidence shows that e-cigarettes are more effective than NRT in supporting people to quit smoking, but the majority of trial evidence comes from people who are motivated to stop smoking rather than people who potentially have no prior intention to quit.^8^ An intervention to treat tobacco dependency in an ED setting using e-cigarettes has not previously been tested.

In this trial we aimed to test the real-world effectiveness of an ED-based brief tailored smoking cessation intervention in comparison with usual care, by comparing continuous smoking abstinence at 6-month follow-up between trial groups.

## Methods

### Trial design

The Cessation of Smoking Trial in the Emergency Department (COSTED) is a two-arm pragmatic, multicentre, parallel-group, individually randomised controlled trial carried out at six UK NHS EDs.^9^ The study protocol has been published^9^ and is available in online supplemental file 1 and the statistical analysis plan is available online.^10^ A full economic evaluation and process evaluation were embedded and will be published separately.

10.1136/emermed-2023-213824.supp1 Supplementary data

### Participants

We recruited adults (aged 18 years or older) who reported smoking tobacco daily, attending the ED for medical treatment or accompanying someone attending for medical treatment. Participants were screened while they were in the ED. People were excluded if they had an expired carbon monoxide (CO) of <8 parts per million (ppm), required immediate medical treatment, were in police custody, had a known allergy to nicotine, were current dual users (defined as daily e-cigarette use), were considered not to have capacity to consent or had already taken part in the trial.

Where the person accompanying an included patient met the inclusion criteria and wished to participate, they were enrolled in a similar way to the patients and assigned to the same treatment group as the patient they accompanied. They were followed up but are not included in the analysis reported in this paper as they were not randomised individually (as per the protocol).

### Randomisation

People who met the inclusion criteria and gave consent were individually randomised (1:1) to the intervention or control groups through a web-based service provided by the Norwich Clinical Trials Unit. This computer-generated randomisation employed varying block sizes and was stratified by the recruitment sites which allowed for concealment of allocation. Due to the participatory nature of the intervention, it was not feasible to blind participants or those delivering the intervention to group allocation.

### Interventions

Participants allocated to the intervention group received an opportunistic smoking cessation intervention undertaken face-to-face in the ED, comprising three elements: (1) brief smoking cessation advice (up to 15 min), (2) the provision of an e-cigarette starter kit plus advice on its use (up to 15 min) and (3) referral to local stop smoking services.

The advice was delivered individually (or with an accompanying person) by a dedicated smoking cessation advisor based in the ED. Protocol-driven^10^ theory-based^11^ smoking cessation advice addressed key aspects of the importance of switching away from tobacco smoking, tailored to the participants’ presenting condition (eg, discussing improved wound healing for patients attending with a laceration). This part of the intervention was a single session undertaken within the ED while patients were waiting to be seen or after discharge.

The e-cigarette starter kit (DotPro, manufactured by Liberty Flights, an independent e-cigarette manufacturer not funded by the tobacco industry) is a ‘pod’ device. The kit included 11 pods (3 tobacco flavoured, 4 berry flavoured and 4 menthol flavoured) of 20 mg/mL nicotine strength. This device was chosen based on in-depth patient and public consultation, considering ease of use, nicotine delivery, satisfaction, price and availability.

Participants were electronically referred to the local stop smoking service which provided routinely available follow-up support. This typically consisted of a telephone call offering support and, if taken up, advice on how to quit and free provision of NRT.

The intervention was delivered by smoking cessation advisors trained specifically for the role. The advisors were either research nurses, research practitioners, ED nurses or healthcare assistants seconded to the trial and received 2.5 days of training. A TIDieR checklist,^12^ logic model and intervention manual are available on the Open Science Framework.^10^

Participants allocated to the control group were given details of local NHS stop smoking services via written material but were not referred directly.

### Procedures

Research assessments were undertaken at baseline and then 1, 3 and 6 months after randomisation. Local site research teams undertook the baseline assessments face-to-face in the ED. Follow-up questionnaires were sent as a link in a text message or email, or by mail with freepost envelopes for return. We attempted to contact all participants who did not respond to the initial request at least twice. Those who reported smoking abstinence at 6 months were invited to undertake a CO reading either at the ED, at a convenient location or remotely by being sent a CO monitor and having a video call with a researcher. Participants were not given details about CO test cut-offs or that it was being used to verify abstinence. All measures except for the CO verification at baseline and 6 months were self-reported. It was not possible to blind outcome assessors to study group.

On completion of the 6-month follow-up questionnaires, participants received a £30 shopping voucher for taking part. A further £30 voucher was offered to participants who reported being smoke-free for providing a CO reading. Participants were, however, unaware they would be offered the additional £30 when completing follow-up questionnaires to avoid it acting as an incentive.

### Outcomes

The primary effectiveness outcome was self-reported continuous smoking abstinence, biochemically validated by CO monitoring at 6 months with a cut-off of <8 ppm (according to the Russell standard).^13^ If smoking status or CO readings could not be obtained, the participant was assumed to be smoking as is agreed practice in smoking cessation trials.^13 14^ Bedfont Micro Smokerlyzers (Bedfont Scientific, Maidstone, UK) were used at baseline and follow-up to measure CO levels. Participants were classified as having been biochemically-verified continuously abstinent if they reported having fewer than six lapses in the last 6 months and gave a CO reading of <8 ppm. Self-reported 7-day abstinence rates were defined as answering ‘no’ to the question “‘Have you smoked a tobacco cigarette in the past 7 days?”

Secondary outcomes were self-reported 7-day point prevalence smoking status at 1 and 3 months, biochemically-validated 7-day point prevalence abstinence at 6 months, number of quit attempts, time to relapse (if applicable), number of cigarettes per day, nicotine dependence,^15^ number of times using an e-cigarette per day, incidence of self-reported dry cough or mouth or throat irritation, motivation to stop smoking,^16^ self-reported use of healthcare services in the last 6 months, self-reported use of smoking cessation services in the last 6 months and quality of life (using the EQ-5D-5L).^17^

Adverse events were self-reported by participants in the follow-up questionnaire in response to the experience of symptoms of a dry cough and throat/mouth irritation. Attendance at hospital was asked about at 1, 3 and 6 months.

Further details of secondary outcomes and adverse events are available in the published protocol.^9^

### Sample size

A sample size of 972 (486 per group) conferred 90% power to detect a difference between a biochemically-confirmed control quit rate of 6.2% and biochemically-confirmed intervention quit rate of 12.2% at the 5% level of significance. This was based on a US trial of an ED smoking cessation intervention using a brief intervention, referral to smoking cessation services and nicotine replacement.^18^ A quit rate of 6.2% was used in the control group based on an average of three studies of unmotivated quitters who received either contact details for stop smoking services or no intervention.^19–21^

### Statistical analysis

The primary outcome measure was compared between the two groups using a binary regression model with a log link to estimate the relative risk and with an identity link to estimate the difference in risk; both models included fixed effects for randomisation group and site. In cases when the convergence failed for the identity link model, a Gaussian model with robust variance was used. Full details of the statistical analysis can be found in the statistical analysis plan online.^10^ Those conducting the analysis were not blinded.

### Patient and public involvement

This trial was initially informed by patient and public involvement (PPI) consultations in three EDs, assessing the acceptability and feasibility of approaching people about smoking cessation. We actively recruited further PPI volunteers who were then involved in trial set-up through advising on study materials, checking Case Report Form burden and advising on language use. A separate PPI panel was recruited to inform intervention components (choice of e-cigarette). We recruited two independent PPI members to be involved in our trial steering group, providing a lay perspective in oversight of the trial. We have shared the results with all our PPI representatives.

## Results

### Participants

**Figure 1:**
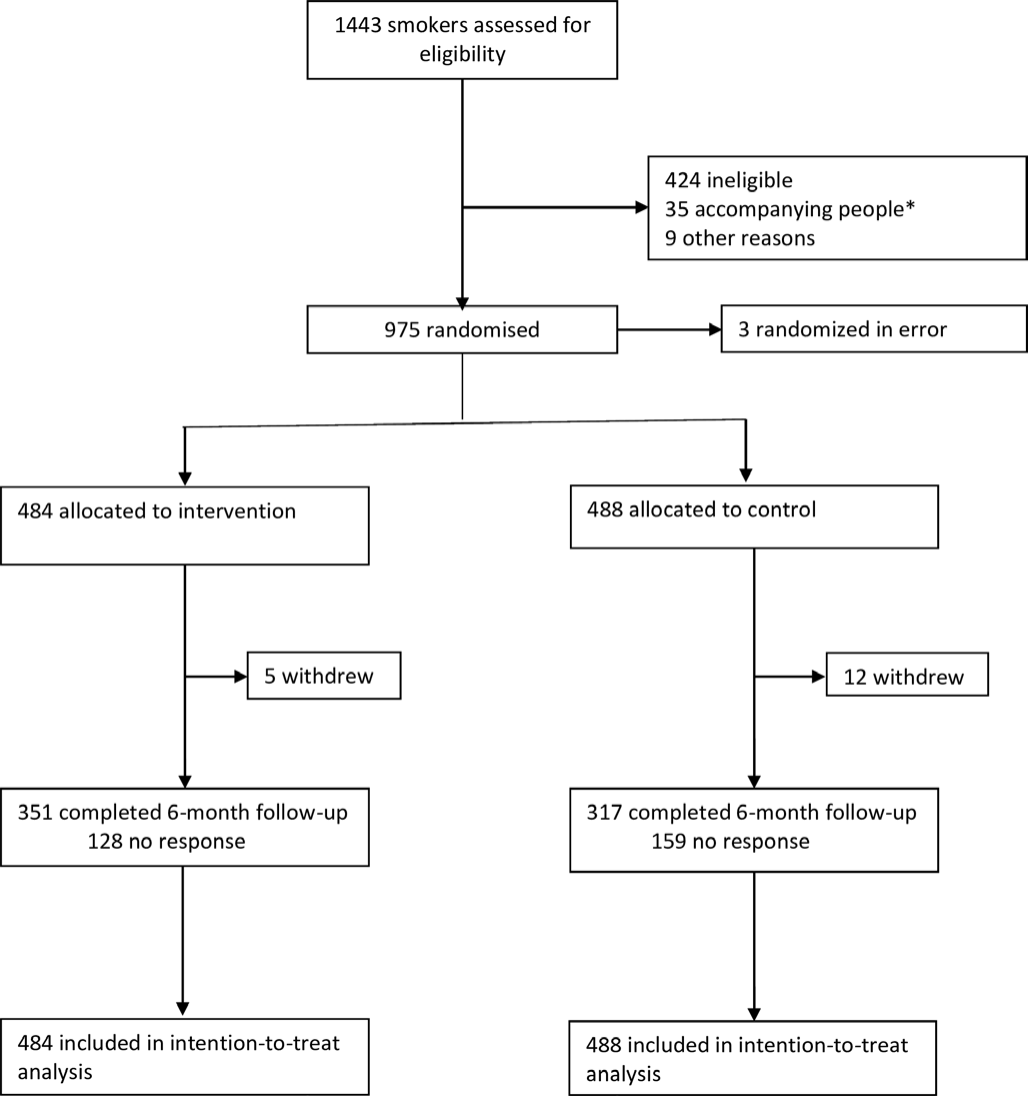
Trial profile. *Where the person accompanying an included patient met the inclusion criteria and wished to participate, they were enrolled in a similar way to the patients and assigned to the same treatment group as the patient they accompanied. They were followed up but are not included in this analysis.

Between January and August 2022 we screened patients in the ED of whom 2888 reported current smoking; 1443 agreed to take part in the trial and were assessed for eligibility and 484 were subsequently randomised to the intervention group and 488 to the control group (figure 1).

The most common reasons for declining to take part were: no reason given (n=409, 29.1%), feeling too unwell (n=296, 21.0%) and not wanting to quit (n=161, 11.4%). The most common reasons for being excluded were providing a CO reading of <8 ppm (n=308, 65.8%), currently using an e-cigarette daily (n=52, 11.1%) and not smoking daily (n=31, 6.6%).

Three participants were found later to be ineligible and were considered post-randomisation exclusions, two due to being randomised twice and one who subsequently reported daily use of an e-cigarette.

There were 5 (1.0%) withdrawals in the intervention group and 12 (2.5%) in the control group. Reasons for withdrawals were no reason given (n=7), wanting the intervention (n=3), did not want to answer the questions (n=6) and reporting a new allergy to nicotine (n=1).

The baseline characteristics of the participants are shown in table 1 and were broadly equivalent across the two groups. The mean deprivation decile was 4.31 in the intervention group and 4.53 in the control group (1=most deprived, 10=least), indicating that participants were generally from more deprived neighbourhoods than average.

**Table 1:** Baseline characteristics of the intention-to-treat population

		Intervention (n=484)	Control (n=488)	Total
Sex	Men	302 (62.4%)	301 (61.7%)	603 (62.0%)
	Women	182 (37.6%)	187 (38.3%)	369 (38.0%)
Mean (SD) age (years)		40.52 (13.58)	40.48 (13.72)	40.50 (13.65)
Ethnic origin	White British	353 (72.9%)	350 (71.7%)	703 (72.3%)
	White – Other	66 (13.6%)	56 (11.5%)	122 (12.6%)
	Black	29 (6.0%)	28 (5.7%)	57 (5.9%)
	South Asian	28 (5.8%)	36 (7.4%)	64 (6.6%)
	Other	7 (1.5%)	17 (3.5%)	24 (2.5%)
	Refused/missing	1 (0.2%)	1 (0.2%)	2 (0.2%)
Mean (SD) deprivation decile		4.31 (2.57) (n=478)	4.53 (2.61) (n=483)	4.42 (2.59) (n=961)
Employment status	Employed	291 (60.1%)	305 (62.5%)	596 (61.3%)
	Unemployed	50 (10.3%)	46 (9.4%)	96 (9.9%)
	Unable to work due to sickness or disability	89 (18.4%)	87 (17.8%)	176 (18.1%)
	Carer, retired or student	52 (10.7%)	50 (10.3%)	102 (10.5%)
	Other	2 (0.4%)	0	2 (0.2%)
Median (IQR) number of cigarettes smoked/day		15 (10–20)	15 (10–20)	15 (10–20)
Mean (SD) motivation to quit score		4.13 (1.58)	4.14 (1.62)	4.13 (1.60)
Mean (SD) age started smoking		16.13 (5.06) (n=484)	15.51 (4.14) (n=487)	15.82 (4.63) (n=971)
Mean (SD) Fagerström test for nicotine dependence score		4.94 (2.27)	4.84 (2.34)	4.89 (2.31)
Use of nicotine replacement therapy in last 3 months		42 (8.7%)	46 (9.4%)	88 (9.1%)
Use of e-cigarettes in last 3 months	Not used	353 (72.9%)	369 (75.6%)	722 (74.3%)
	Once a month or less	39 (8.1%)	55 (11.3%)	94 (9.7%)
	On 2–4 days a month	36 (7.4%)	20 (4.1%)	56 (5.8%)
	On 2–3 days a week	26 (5.4%)	23 (4.7%)	49 (5.0%)
	On 5–6 days a week	30 (6.2%)	21 (4.3%)	51 (5.3%)
	Daily	0	0	0
Lives with other smoker(s)		214 (44.2%)	185 (37.9%)	399 (41.1%)
Recruitment by site	Site 1	199 (41.1%)	201 (41.2%)	400 (41.2%)
	Site 2	84 (17.4%)	84 (17.2%)	168 (17.3%)
	Site 3	54 (11.2%)	53 (10.9%)	107 (11.0%)
	Site 4	74 (15.3%)	76 (15.6%)	150 (15.4%)
	Site 5	50 (10.3%)	50 (10.3%)	100 (10.3%)
	Site 6	23 (4.8%)	24 (4.9%)	47 (4.8%)

### Primary outcome

Biochemically-verified self-reported continuous abstinence at 6 months was 7.2% (35/484) in the intervention group and 4.1% (20/488) in the control group (relative risk (RR) 1.76 (95% CI 1.03 to 3.01), risk difference 3.3% (95% CI 0.3% to 6.3%)).

In total, 351 (72.5%) participants in the intervention group and 317 (65.0%) in the control group reported their smoking status at 6 months (figure 1). Of those who reported continuous abstinence, 35/122 (28.7%) in the intervention group and 20/64 (31.3%) in the control group went on to have their abstinence biochemically verified. Sixty-eight participants in the intervention group and 32 in the control group declined to provide a CO reading, and 19 in the intervention group and 12 in the control group had a CO reading ≥8 ppm.

The online supplemental material shows a sensitivity analysis for the assumption that those who did not respond or did not provide a CO reading were still smoking. Provided the dropouts have <0.2 times the odds of being abstinent than those who remain, the intervention is statistically significant. Even under the assumption that the dropouts are equally likely to smoke as those who remain, the estimated adjusted OR is still larger than 1.5 (1.56, 95% CI 0.88 to 2.76), but no longer statistically significant.

10.1136/emermed-2023-213824.supp2 Supplementary data

### Secondary outcomes

Self-reported 7-day abstinence at 6 months was 23.3% (113/484) in the intervention group and 12.9% (63/488) in the control group (RR 1.80 (95% CI 1.36 to 2.38); p<0.0001). Table 2 shows the abstinence rates at all time points.

**Table 2:** Abstinence rates at different time points

	Intervention (n=484)	Control (n=488)	Absolute difference (95% CI)	P value	Relative risk (95% CI)	P value
Primary outcome: biochemically validated self-reported continuous smoking abstinence at 6 months	35 (7.2%)	20 (4.1%)	3.3 (0.3 to 6.3)	0.032	1.76 (1.03 to 3.01)	0.038
Self-reported 7-day abstinence at 1 month	94 (19.4%)	49 (10.0%)	9.0 (4.9 to 13.7)*	<0.0001	1.92 (1.39 to 2.64)	<0.0001
Self-reported 7-day abstinence at 3 months	113 (23.3%)	58 (11.9%)	11.3 (6.6 to 16.1)	<0.0001	1.97 (1.47 to 2.63)	<0.0001
Self-reported 7-day abstinence at 6 months	113 (23.3%)	63 (12.9%)	10.6 (5.86 to 15.41)	<0.0001	1.80 (1.36 to 2.38)	<0.0001

*Based on Gaussian model with robust variances due to lack of convergence.

The number needed to treat to achieve biochemically-validated smoking continuous abstinence at 6 months was 30 (95% CI 16 to 343) and for self-reported abstinence at 6 months it was 9 (95% CI 6 to 11).

At 6 months the median (IQR) number of quit attempts was 2 (1–4) in the intervention group and 1 (0–3) in the control group (p<0.0001). Of those who responded, the number of participants using an e-cigarette daily at 6 months was 39.4% (125/317) in the intervention group and 17.5% (53/303) in the control group (table 3). The number reporting not having used an e-cigarette in the past 6 months was 14.8% (47/317) in the intervention group and 54.5% (165/303) in the control group.

**Table 3:** Secondary outcome measures

	Intervention	Control	Absolute difference (95% CI)	P value
Number of cigarettes smoked at 6 months, median (IQR)	0 (0–10) n=314	10 (0–15) n=283	−8 (−10.41 to 5.59)	<0.0001
Number of quit attempts, median (IQR)	2 (1–4) n=183	1 (0–3) n=229		<0.0001
Number of times using an e-cigarette per day at 6 months, median (IQR)	5 (0–10) n=176	0 (0–3) n=239	5 (4.04 to 5.96)	<0.0001
Frequency of e-cigarette use in past 6 months, n (%)				<0.0001
Not used	47 (14.8%)	165 (54.5%)		
Once a month or less	39 (12.3%)	24 (7.9%)		
On 2–4 days a month	32 (10.1%)	25 (8.3%)		
On 2–3 days a week	52 (16.4%)	23 (7.6%)		
On 5–6 days a weeks	22 (6.9%)	13 (4.3%)		
Daily	125 (39.4%)	53 (17.5%)		
Dry cough in last week, at 6 months, median (IQR), n (%)	1 (1–2) n=310	1 (1–3) n=292		0.344
1 (not at all)	174 (56.1%)	154 (52.7%)		
2	60 (19.4%)	57 (19.5%)		
3	46 (14.8%)	47 (16.1%)		
4	17 (5.5%)	22 (7.5%)		
5 (extremely)	13 (4.2%)	12 (4.1%)		
Throat/mouth irritation in last week, at 6 months, median (IQR), n (%)	1 (1–2) n=310	1 (1–2) n=293		0.117
1 (not at all)	206 (66.5%)	176 (60.1%)		
2	46 (14.8%)	49 (16.7%)		
3	31 (10.0%)	41 (14.0%)		
4	17 (5.5%)	19 (6.5%)		
5 (extremely)	10 (3.2%)	8 (2.7%)		
Motivation to stop smoking	4 (3–5) n=177	4 (2–5) n=227		0.432
Mean (SD) Fagerström test for nicotine dependence score	3.70 (2.21) n=185	4.17 (2.24) n=224	−0.51 (−0.95 to −0.07)	0.022

### Safety

The number of participants reporting serious adverse events was 5.2% (25/484) in the intervention group and 5.1% (25/488) in the control group (table 4). None were related to the intervention.

**Table 4:** Adverse event by type

	Intervention (n=484)	Control (n=488)
Serious adverse events (≥1)*, n (%)	25 (5.2%)	25 (5.1%)
Adverse events (≥1), n (%)		
Throat/mouth irritation (extreme)	10 (3.2%)	8 (2.7%)
	n=310	n=293
Dry cough (extreme)	13 (4.2%)	12 (4.1%)
	n=310	n=292

*All serious adverse events resulting in hospitalisation over the study period.

## Discussion

### Principal findings

In this trial adults attending the ED who smoked and received the intervention of brief advice, an e-cigarette starter kit and referral to stop smoking services were statistically significantly more likely to achieve sustained smoking abstinence than those who received signposting to stop smoking services alone. The biochemically-verified quit rate was not as high as the assumptions underpinning the power calculation; however, the difference found achieved statistical significance, with the potential to impact on population smoking prevalence. There was a much larger difference in self-reported abstinence compared with the power calculation, which may indicate that the biochemically-verified quit rate is an underestimate of the true effect of the intervention.

### Comparison with previous studies

These results strengthen previous findings that ED-based smoking cessation interventions are effective.^7^ To our knowledge, the 6-month self-reported quit rate is the highest reported by any ED-based smoking cessation intervention trial to date. As the first ED-based trial to include an e-cigarette starter kit as part of the intervention, this suggests that the e-cigarette itself, in addition to brief advice, may have contributed to the size of the effect. The findings are in keeping with existing evidence that e-cigarettes are effective in aiding smoking cessation,^8 22^ but are novel as this is the first trial to use them opportunistically to support abstinence in those who smoke and are accessing healthcare services, but who are not actively seeking help to quit.

Of people who smoke attending the ED, half were willing to take part in the trial, indicating that the ED represents an acceptable location for smoking cessation intervention and therefore offers a valuable opportunity to engage those who smoke who are not currently seeking to quit.

### Strengths and limitations

The strengths of this study include: its large sample size; it was inclusive, being delivered across multiple UK centres recruiting a diverse population; it used an objective primary outcome measure; inclusion criteria were broad to ensure generalisability; it had a robust study design with appropriate randomisation and allocation concealment and the trial had a pragmatic design with an intervention that should be easy to replicate in day-to-day practice assuming it is appropriately resourced.

A limitation of the study was that control participants did not simply receive a leaflet signposting them to stop smoking services, as in order to collect the data needed from the control group there was a discussion with researchers that may have affected smoking behaviour. They underwent CO breath testing, were asked extensive questions about their smoking, received written information on stop smoking services and were asked their smoking status three times over the follow-up period. This may have caused a higher quit rate in the control group compared with true usual care (which is likely to have no mention of quitting smoking) and therefore potentially underestimates the impact of the intervention.

Successfully encouraging our trial participants to submit a CO reading at 6 months proved to be very challenging. This may in part be due to the transient and sometimes chaotic nature of the lives of many ED attendees, the large geographical catchment area of participating EDs and transportation complexities. Thus, the biochemically confirmed cessation rates (while statistically significant) may underestimate the true effect size. Equally, it is possible that being part of the intervention group encouraged more of those to provide biochemical confirmation, although our biochemical confirmation findings mirrored our self-report findings and the percentage of CO verifications at 6 months was similar across the intervention and control groups. While the biochemically confirmed quit rates in the intervention and control groups were not as large as the power calculation had been based on, the self-reported continuous abstinence rate was much larger. The difference is likely a result of the difficulty with collecting CO readings. There was a difference in response rate between the intervention and control groups. As is convention in smoking cessation trials, we assumed that those who did not respond were still smoking;^23^ however, this assumption may be conservative and has been examined in a sensitivity analysis in the online supplemental material. The challenges achieving biochemical verification and the differences in response rates between the groups are limitations which arise from this being a pragmatic trial which attempted to replicate real life. This is in keeping with other ED-based smoking cessation trials which attempted to biochemically verify smoking status with loss to follow-up rates of around 30% and biochemical verification of those reporting abstinence of around 50%.

### Policy implications

This trial has demonstrated the effectiveness of a simple, opportunistic and acceptable intervention in a real-world setting with no serious adverse effects. We consider that this could be rolled out to reach a large proportion of current smokers, although dedicated staff are clearly needed to deliver the intervention so as not to burden clinical staff. Those attending EDs are generally from more deprived communities and more likely to smoke than the general population.^5 6^ Therefore, this intervention has the potential to address health inequalities that arise from disparities in smoking rates between different socioeconomic groups.^3^

Given high accessibility to an at-risk population, future research might explore the use of EDs as a location to support people to change other behaviours such as excess alcohol use or low physical activity.

## Conclusion

In this study of adults who smoke and who were attending the ED, an intervention comprising brief advice, provision of an e-cigarette starter kit and referral to stop smoking services resulted in significantly increased sustained smoking abstinence 6 months later compared with those signposted to stop smoking services. Providing smoking cessation support in the ED should be considered to reach groups of the population that may not routinely engage with stop smoking services but have the most to gain from stopping smoking.

## Data Availability

Data are available upon reasonable request. The protocol, consent form, statistical analysis plan, medical ethics committee approvals, training materials and other relevant study materials are available online at https://osf.io/8hbne/. Deidentified participant data will be made publicly available within 3 months at the above address.
