# Supplementary material for: Cessation of Smoking Trial in the Emergency Department (COSTED): a multicentre randomised controlled trial
Source: Emerg Med J. 2024 Mar 26;41(5):276–82. doi: 10.1136/emermed-2023-213824 (PMC11041600; doi:10.1136/emermed-2023-213824)
Supplement: Supplementary data [file emermed-2023-213824supp001.pdf]

DocuSign Envelope ID: 77E6F4CA-8F41-42B4-8991-9A76FB043639

CoSTED

Cessation of Smoking Trial in the Emergency Department

Protocol Version	Version 2.0
Protocol Date	22nd September 2021
Sponsor	Norfolk and Norwich University Hospitals NHS Foundation Trust (NNUH)
Trial registration	ClinicalTrial.gov: NCT04854616
NRES Number	21/SC/0288

Pope I, *et al. Emerg Med J* 2024; 41:276–282. doi: 10.1136/emermed-2023-213824

DocuSign Envelope ID: 77E6F4CA-8F41-42B4-8991-9A76FB043639

Authorisation: Co-Chief Investigator

Name	Ian Pope
Role	Co-Chief Investigator
Signature	DocuSigned by: Ian Pope E5A8C444E55845E...
Date	09 November 2021

Authorisation: Co-Chief Investigator

Name	Caitlin Notley
Role	Co-Chief Investigator
Signature	DocuSigned by: Caitlin Notley 52E681A55B164A8...
Date	14 November 2021

Authorisation: Sponsor

Name	Michael Sheridan
Role	Sponsor Representative
Signature	DocuSigned by: Michael Sheridan 572111C0F919447...
Date	09 November 2021

Authorisation: CTU Director or Representative

Name	Matthew Hammond
Role	NCTU Deputy Director
Signature	DocuSigned by: Matthew Hammond 7153BBAFE90F42E...
Date	09 November 2021

Authorisation: Trial Statistician

Name	Allan Clark
Role	Lead Statistician
Signature	DocuSigned by: Allan Clark AC8ACE6AEBA34CA...

DocuSign Envelope ID: 77E6F4CA-8F41-42B4-8991-9A76FB043639

Date	09 November 2021

Authorisation: Trial Health Economist

Name	Steve Parrott
Role	Lead Health Economist
Signature	DocuSigned by: Steve Parrott 9CA02D4D6D3D432...
Date	12 November 2021

DocuSign Envelope ID: 77E6F4CA-8F41-42B4-8991-9A76FB043639

Table of Contents

1 Administrative information 8
1.1 Compliance 8
1.2 Sponsor 9
1.3 Structured trial summary 10
1.4 Roles and responsibilities 13
1.4.1 Protocol contributors 13
1.4.2 Role of trial sponsor and funders 13
1.4.3 Trial Team 13
1.4.4 Trial Management Group 13
1.4.5 Trial Steering Committee 14
1.4.6 Data Monitoring Committee 14
2 Trial Diagram 15
3 Abbreviations 16
4 Introduction 18
4.1 Background and Rationale 18
4.1.1 Explanation for choice of comparators 20
4.2 Objectives 20
4.3 Trial Design 21
5.0 Methods 21
5.1 Site Selection 21
5.1.1 Study Setting 21
5.1.2 Site/Investigator Eligibility Criteria 21
5.1.2.1 Principal Investigator's (PI) Qualifications and Agreements 22
5.1.2.2 Resourcing at site 22
5.2 Site approval and activation 22
5.3 Participants 22
5.3.1 Eligibility Criteria 22
5.3.1.1 Participant Inclusion Criteria 23
5.3.1.2 Participant Exclusion Criteria 23
5.3.1.3 Procedure for accompanying person 23
5.3.1.4 Procedure if admission is required or participant requires immediate medical treatment 23
5.3.1.5 Eligibility Criteria for Individuals Performing the Interventions 23
5.3.1.6 Co-enrolment Guidance 24

DocuSign Envelope ID: 77E6F4CA-8F41-42B4-8991-9A76FB043639

5.3.1.7 Screening Procedures and Pre-randomisation Investigations 24
5.4 Internal Pilot 24
5.5 Interventions 25
5.5.1 Arm A – Treatment as Usual (TAU) 25
5.5.2 Arm B – CoSTED Intervention 25
5.5.2.1 Intervention components 25
5.5.2.2 Training for those delivering the intervention 26
5.5.3 Accountability 27
5.5.4 Compliance and Adherence 27
5.5.5 Crossover between trial groups 28
5.5.6 Concomitant Care 28
5.5.7 Protocol Intervention Discontinuation 28
5.6 Outcomes 28
5.6.1 Primary Outcomes 28
5.6.2 Secondary Outcomes 29
5.6.3 Process Evaluation 29
5.6.3.1 Managing distress or harm following participant interviews 30
5.7 Participant Timeline 31
5.7.1 Patient Assessments 32
5.7.1.1 Screening 32
5.7.1.2 Baseline 32
5.7.1.3 Smoking cessation advice 32
5.7.1.4 1-month and 3 month Follow-Up 32
5.7.1.5 6 Month Follow-up 33
5.7.2 Early Stopping of Follow-up 33
5.7.3 Participant Transfers 34
5.7.4 Loss to Follow-up 34
5.7.5 Trial Closure 34
5.8 Sample Size 34
5.9 Recruitment and Retention 34
5.9.1 Recruitment 34
5.9.2 Retention 35
5.10 Assignment of Intervention 35
5.10.1 Allocation 35

DocuSign Envelope ID: 77E6F4CA-8F41-42B4-8991-9A76FB043639

5.10.1.1 Sequence generation 35
5.10.1.2 Allocation concealment mechanism 35
5.10.1.3 Allocation Implementation 36
5.10.2 Blinding 36
5.11 Data Collection, Management and Analysis 36
5.11.1 Data Collection Methods 36
5.11.2 Data Management 37
5.11.3 Non-Adherence and Non-Retention 38
5.11.4 Statistical Methods 38
5.11.4.1 Outcomes 39
5.11.4.2 Statistical Analysis Plan 39
5.11.4.3 Additional Analyses 40
5.11.4.4 Analysis Population 40
5.11.4.5 Missing Data 40
5.11.5 Economic evaluations 40
5.11.5.1 Health Economics Analysis Plan 41
5.11.5.2 Within-trial analysis 41
5.11.5.3 Model-based analysis 41
5.11.6 Analysis of Qualitative Information 42
5.11.7 Analysis of Observational Information 42
5.12 Data Monitoring 42
5.12.1 Data Monitoring Committee (DMC) 42
5.12.2 Interim Analyses 42
5.12.3 Data Monitoring for Harm 42
5.12.3.1 Adverse Event/Reaction Reporting 42
5.12.3.2 Procedure for those with potential contraindications 43
5.12.3.3 Identifying Adverse Events 43
5.12.3.4 Investigator responsibilities relating to safety reporting 44
5.12.3.4.1 Seriousness assessment 44
5.12.3.4.2 Causality 44
5.12.3.4.3 Expectedness 44
5.12.3.5 Notifications 45
5.12.3.5.1 Notifications by the Investigator to NCTU 45
5.12.3.5.2 NCTU responsibilities 45
5.12.4 Quality Assurance and Control 45

DocuSign Envelope ID: 77E6F4CA-8F41-42B4-8991-9A76FB043639

5.12.4.1 Risk Assessment ... 45
5.12.4.2 Central Monitoring at NCTU ... 46
5.12.4.3 On-site Monitoring ... 46
5.12.4.3.1 Direct access to participant records ... 46
5.12.4.4 Trial Oversight ... 46
5.12.4.4.1 Core Trial Team (TT) ... 46
5.12.4.4.2 Trial Management Group (TMG) ... 47
5.12.4.4.3 Independent Trial Steering Committee (TSC) ... 47
5.12.4.4.4 Independent Data Monitoring Committee (IDMC) ... 47
5.12.4.4.5 Patient and Public Involvement (PPI) ... 47
5.12.4.4.6 Trial Sponsor ... 47
6 Ethics and Dissemination ... 48
6.1 Research Ethics and Health Research Authority Approval ... 48
6.2 Competent Authority Approvals ... 48
6.3 Other Approvals ... 48
6.4 Amendments ... 48
6.5 Consent ... 49
6.5.1 Consent in Ancillary Studies ... 49
6.6 Confidentiality ... 49
6.7 Declaration of Interests ... 49
6.8 Indemnity ... 49
6.9 Finance ... 49
6.10 Archiving ... 50
6.11 Access to Data ... 50
6.12 Ancillary and Post-trial Care ... 50
6.13 Publication Policy ... 50
6.13.1 Trial Results ... 50
6.13.2 Dissemination ... 50
6.13.3 Authorship ... 51
6.13.4 Reproducible Research ... 51
7 Ancillary Studies ... 51
8 Protocol Amendments ... 52
9 References ... 53

DocuSign Envelope ID: 77E6F4CA-8F41-42B4-8991-9A76FB043639

1 Administrative information

This document was constructed using the Norwich Clinical Trials Unit (NCTU) Protocol template Version 4. It describes the CoSTED trial, sponsored by The Norfolk and Norwich University Hospitals NHS Foundation Trust (NNUH) and co-ordinated by NCTU.

It provides information about procedures for entering participants into the trial, and provides sufficient detail to enable: an understanding of the background, rationale, objectives, trial population, intervention, methods, statistical analyses, ethical considerations, dissemination plans and administration of the trial; replication of key aspects of trial methods and conduct; and appraisal of the trial's scientific and ethical rigour from the time of ethics approval through to dissemination of the results. The protocol should not be used as an aide-memoire or guide for the treatment of other patients. Every care has been taken in drafting this protocol, but corrections or amendments may be necessary. These will be circulated to registered investigators in the trial. Sites entering participants for the first time should confirm they have the correct version through a member of the trial team at NCTU.

NCTU supports the commitment that its trials adhere to the SPIRIT guidelines. As such, the protocol template is based on the Standard Protocol Items: Recommendations for Interventional Trials (SPIRIT) 2012 Statement for protocols of clinical trials (Chan et al., 2013a). The SPIRIT Statement Explanation and Elaboration document (Chan et al., 2013b) can be referred to, or a member of NCTU Protocol Review Committee can be contacted for further detail about specific items.

1.1 Compliance

The trial will be conducted in compliance with the approved protocol, the Declaration of Helsinki (2008), the principles of Good Clinical Practice (GCP) as laid down by the Commission Directive 2005/28/EC with implementation in national legislation in the UK by Statutory Instrument 2004/1031 and subsequent amendments, the UK Data Protection Act, and the UK Policy Framework for Health and Social Care Research, the Mental Capacity Act 2005, and other national and local applicable regulations. Agreements that include detailed roles and responsibilities will be in place between participating sites and NCTU.

An e-cigarette can be defined as 'an electronic vaping device that is handheld and produces, for inhalation, an aerosol formed by heating an e-liquid using a battery-powered heating coil' (Addiction Ontology 2021). E cigarettes are consumer products regulated by the Tobacco Products Directive 2014/40/EU (TPD); they are not considered an investigational medicinal product (IMP) in health/clinical research. As this trial is not a Clinical Trial of an Investigational Medicinal Product (IMP) as defined by the EU Directive 2001/20/EC a Clinical Trial Agreement (CTA) is not required to undertake this trial in the UK.

Participating sites will inform NCTU as soon as they are aware of a possible serious breach of compliance, so that NCTU can fulfil its requirement to report the breach if necessary, within the timelines specified in the UK Clinical Trials Regulations (currently 7 days). For the purposes of this regulation a 'serious breach' is one that is likely to affect to a significant degree:

- The safety or physical or mental integrity of the subjects in the trial, or
- The scientific value of the trial.

DocuSign Envelope ID: 77E6F4CA-8F41-42B4-8991-9A76FB043639

1.2 Sponsor

The NNUH is the trial sponsor and has delegated responsibility for the overall management of the CoSTED trial to the Chief Investigators (CI) and NCTU. Queries relating to sponsorship of this trial should be addressed to the Chief Investigators or via the trial team.

DocuSign Envelope ID: 77E6F4CA-8F41-42B4-8991-9A76FB043639

1.3 Structured trial summary

Primary Registry and Trial Identifying Number	ClinicalTrial.gov: NCT04854616
Date of Registration in Primary Registry	22nd April 2021
Secondary Identifying Numbers	• Sponsor ID: 123-08-20<br>• IRAS: 263674
Source of Monetary or Material Support	National Institute for Health Research – Health Technology Assessment [NIHR129438]
Sponsor	The Norfolk and Norwich University Hospitals NHS Foundation Trust (NNUH)
Contact for Public Queries	costed@uea.ac.uk
Contact for Scientific Queries	Dr Ian Pope<br>Emergency Department<br>Norfolk and Norwich University Hospital NHS Foundation Trust<br>Colney Lane, Norwich, NR4 7UY.<br>Email: i.pope@uea.ac.uk<br>Telephone: 01603 597191
Short Title or Acronym	CoSTED
Scientific Title	Cessation of Smoking Trial in the Emergency Department
Countries of Recruitment	England, Scotland
Health Condition(s) or Problem(s) Studied	People who smoke attending an Emergency Department for any reason. The intervention is to support smoking cessation.
Intervention(s)	Intervention: Opportunistic smoking cessation intervention comprising brief smoking cessation advice, the provision of an e-cigarette starter kit and training in its use and referral to stop smoking services (SSS). Following trial specific training up to 15 minutes of advice will be delivered by a smoking cessation advisor who will follow a protocol. Advice will address key aspects of the importance of quitting smoking, tailored to the patient where possible. Advice of up to 15 minutes will also be offered about how to use an e-cigarette efficiently to satisfy nicotine cravings, alongside the referral to local SSS.<br><br>Control: Usual care plus signposting to NHS smoking cessation services through provision of written information about local services.
Key Inclusion and Exclusion Criteria	Study population:<br><br>Adults ≥18 years attending the Emergency Department (ED)

DocuSign Envelope ID: 77E6F4CA-8F41-42B4-8991-9A76FB043639

	who are current daily tobacco smokers, where smoking is defined as self-reported smoking of at least one-cigarette per day.<br><br>*Inclusion criteria:*<br><br>1. Adult ≥ 18 years<br>2. Current daily tobacco smoker (self-reporting smoking of at least one cigarette per day).<br>3. Attending the ED for medical treatment (or accompanying a patient attending for medical treatment).<br>4. Submitting an expired carbon monoxide (CO) breath test reading of ≥8 parts per million (ppm).<br><br>*Exclusion criteria*:<br><br>1. Requiring immediate medical treatment as defined by the treating clinician, therefore unavailable.<br>2. In police custody.<br>3. A known history of allergy to nicotine replacement products.<br>4. Currently defined as a dual user (already using an e-cigarette daily as well as smoking tobacco daily).<br>5. Without the capacity to give informed consent for participation in the study<br>6. Have taken part in the CoSTED trial already
Study Type	A two-group, multi-centre, pragmatic, individually randomised, controlled trial with an embedded pilot, process evaluation and economic evaluation.<br>Note: this is a non-CTIMP trial.
Date of First Enrolment	Anticipated: 01 October 2021
Target Sample Size	n = 972
Primary Outcome(s)	The primary effectiveness outcome will be self-reported continuous smoking abstinence, biochemically validated by carbon monoxide monitoring at 6 months with cut off of ≥8ppm.
Key Secondary Outcomes	1. Secondary outcomes to be analysed at 6 months, will be:<br>• 7-day point prevalence abstinence<br>• Number of quit attempts<br>• Time to relapse (if applicable)<br>• Number of cigarettes per day<br>• Nicotine dependence<br>• Number of times using an e-cigarette per day<br>• Incidence of self-reported dry cough and mouth/throat irritation<br>• Carbon monoxide concentration for those who report smoking cessation<br>• Motivation to Stop Smoking<br>2. Intervention-related costs, self-reported use of health

DocuSign Envelope ID: 77E6F4CA-8F41-42B4-8991-9A76FB043639

	services and smoking cessation services/aids over 6 months, and health-related quality of life (EQ-5D-5L) will be collected for health economic analysis.<br>3. Self-reported smoking status and adverse events/reactions will be collected at 1 and 3 months.<br>4. Qualitative interviews with a sample of trial participants will follow the trial.<br>5. Qualitative interviews with smoking cessation advisors will follow the trial.

1.4 Roles and responsibilities

These membership lists are correct at the time of writing; please see terms of reference documentation in the TMF for current lists.

1.4.1 Protocol contributors

Name	Affiliation	Role
Ian Pope	NNUH	Co-Chief Investigator (Emergency Department Expertise)
Caitlin Notley	UEA	Co-Chief Investigator (Smoking Cessation Expertise)
Allan Clark	UEA	Co-Applicant, Lead Statistician
Martin Pond	UEA	Head of Data Management
Emma Ward	UEA	Research Fellow
Pippa Belderson	UEA	Senior Research Associate
Steve Parrott	University of York	Lead Health Economist
Jinshuo Li	University of York	Research Fellow (Health Economics)
Lucy Clark	NCTU	Trial Manager
Mei-See Man	NCTU	Senior Trial Manager

1.4.2 Role of trial sponsor and funders

Name	Affiliation	Role
Julie Dawson	NNUH	Sponsor's Representative
Julie Dawson	NNUH	NHS Host Representative
Member of Team	NIHR (HTA)	Funder contact

1.4.3 Trial Team

Name	Affiliation	Role and responsibilities
Ian Pope	NNUH	Co-Chief Investigator
Caitlin Notley	UEA	Co-Chief Investigator
Emma Ward	UEA	Research Fellow
Pippa Belderson	UEA	Senior Research Associate
Gemma Richardson	UEA	Trial Assistant
Mei-See Man	UEA	NCTU Senior Trial Manager
Lucy Clark	UEA	NCTU Trial Manager

1.4.4 Trial Management Group

Name	Affiliation	Role and responsibilities
Ian Pope	NNUH	TMG Co-Chair
Caitlin Notley	UEA	TMG Co-Chair
Allan Clark	UEA	Member
Sanjay Agrawal	Leicester Royal Infirmary	Member
Ben Bloom	Royal London Hospital	Member

DocuSign Envelope ID: 77E6F4CA-8F41-42B4-8991-9A76FB043639

Steve Parrott	The University of York	Member
Linda Bauld	The University of Edinburgh	Member
Richard Holland	University of Leicester	Member
Adrian Boyle	Addenbrooke's Hospital	Member
(Michael) Geraint Morris	Homerton University Hospital	Member
Sarah Gentry	UEA	Member
Alasdair Gray	The University of Edinburgh	Member
Tim Coats	University of Leicester	Member
PPI	TBC	Member
PPI	TBC	Member
Julie Dawson	NNUH	Sponsor
Matt Hammond	NCTU	NCTU Deputy Director
Martin Pond (or rep)	NCTU	NCTU Head of Data Management
Pippa Belderson	UEA	Senior Research Associate
Emma Ward	UEA	Research Fellow
Mei-See Man	NCTU	NCTU Senior Trial Manager
Lucy Clark	NCTU	NCTU Trial Manager

1.4.5 Trial Steering Committee

Name	Affiliation	Role and responsibilities
Ian Pope	NNUH	Co-Chief Investigator
Caitlin Notley	UEA	Co-Chief Investigator
Allan Clark	UEA	Lead Statistician
Deb Smith	N/A	Independent PPI Member
Steve Goodacre	University of Sheffield	Independent Clinical Member (Chair)
Sarah Jackson	University College London	Independent Methodological Member
Francesca Pesola	Queen Mary University of London	Independent Statistical Member
Paul McCrone	University of Greenwich	Independent Health Economist
Julie Dawson	NNUH	Sponsor

1.4.6 Data Monitoring Committee

Name	Affiliation	Role and responsibilities
Ian Pope	NNUH	Co-Chief Investigator
Caitlin Notley	UEA	Co-Chief Investigator
Gary Abel	University of Exeter	Independent Statistician (Chair)
Jamie Brown	University College London	Independent Member
Kirsty Challen	Lancashire Teaching Hospitals NHS FT	Independent Clinician
Chief Investigator	UEA	Non-independent Trial Team member

DocuSign Envelope ID: 77E6F4CA-8F41-42B4-8991-9A76FB043639

2 Trial Diagram

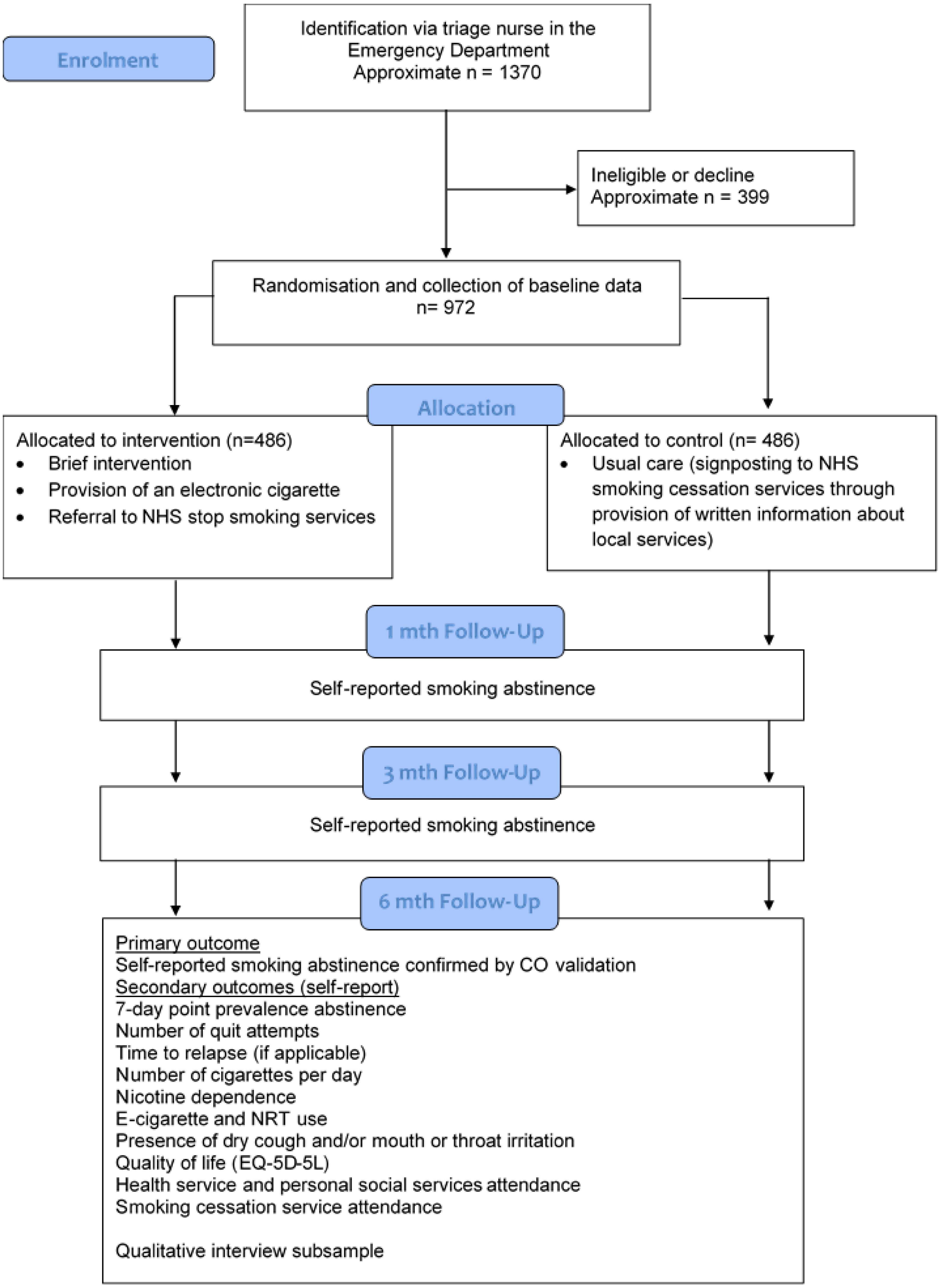

DocuSign Envelope ID: 77E6F4CA-8F41-42B4-8991-9A76FB043639

3 Abbreviations

AE	Adverse Event
AR	Adverse Reaction
CI	Chief Investigator
CO	Carbon Monoxide
CRF	Case Report Form
CTA	Clinical Trial Agreement
DMC	Data Management Committee
ED	Emergency Department
EU	European Union
FDA	(US) Food and Drug Administration
FWA	Federal Wide Assurance
GCP	Good Clinical Practice
HRA	Health Research Authority
HUH	Homerton University Hospital (Site)
ICH	International Conference on Harmonisation
ISF	Investigator Site File
ITT	Intention to Treat
LRI	Leicester Royal Infirmary (Site)
NAE	Notifiable Adverse Event
NCTU	Norwich Clinical Trials Unit
NHS	National Health Service
NRT	Nicotine Replacement Therapy
NNUH	Norfolk and Norwich University Hospitals NHS Foundation Trust
PI	Principal Investigator
PIN	Participant Identification Number
PIS	Participant Information Sheet
PPI	Patient and Public Involvement
PPM	Parts Per Million
PROMS	Patient Reported Outcome Measures
PSS	Personal Social Services
QA	Quality Assurance
QALY	Quality Adjusted Life Year
QC	Quality Control
QMMP	Quality Management and Monitoring Plan
R&D	Research and Development
REC	Research Ethics Committee
RIE	Royal Infirmary Edinburgh (Site)
RLH	Royal London Hospital (Site)
SAE	Serious Adverse Event
SAP	Statistical Analysis Plan
SAR	Serious Adverse Reaction
SF	Site File
SPC	Summary of Product Characteristics
SSA	Site Specific Approval
SUSAR	Suspected Unexpected Serious Adverse Reaction
TMF	Trial Master File
TMG	Trial Management Group
TMT	Trial Management Team

DocuSign Envelope ID: 77E6F4CA-8F41-42B4-8991-9A76FB043639

ToR	Terms of Reference
TSC	Trial Steering Committee
TT	Trial Team
UEA	University of East Anglia

DocuSign Envelope ID: 77E6F4CA-8F41-42B4-8991-9A76FB043639

4 Introduction

4.1 Background and Rationale

Problem: Smoking cessation interventions usually target people who demonstrate initial motivation to quit. However, evidence suggests that 'unmotivated quitters' (i.e. those not actively seeking support) are also aware of the health risks of smoking and many of them are willing to consider quitting (Chan et al., 2011). Smoking rates are four times higher in the most disadvantaged populations compared to the most affluent (Office for National Statistics 2018a). Those with mental health problems are far more likely to smoke (Royal College of Physicians, Royal College of Psychiatrists, 2013). Both groups are also more likely to attend the Emergency Department (ED) (Keane et al., 2007; Rudge et al., 2013). Indeed, our patient and public involvement (PPI) work, undertaken in 2019, showed that approximately 24% of ED attendees were current smokers, many more than in the wider population estimate of 15% in that same year (Smoking in England, 2021, http://www.smokinginengland.info/). This makes the ED an ideal opportunity to screen for smoking status and deliver a smoking cessation intervention.

The NHS Long Term Plan (NHS, 2019) and a recent Royal College of Physicians report (Royal College of Physicians, 2018) suggest that everything possible should be done to 'make every contact count' and offer smoking cessation support to current smokers accessing NHS care. There is good evidence that smokers in the ED are willing to consider quitting (Boudreaux et al., 2005). Previous public health interventions in the ED have failed due to time pressures on ED staff delivering those interventions (Drummond et al. 2014). This problem is addressed by having a dedicated advisor in the ED delivering smoking cessation advice without other time pressures.

If the trial is successful in increasing smoking cessation rates and demonstrates cost effectiveness of the intervention then we envisage that the advisor will be based in the ED full time, during usual working hours, delivering smoking cessation advice, combined with nicotine and behavioural replacement in the form of an e-cigarette, to all patients who attend and are current smokers.

Importance: Population level smoking prevalence is currently around 15% in Great Britain (Office for National statistics 2018b) but smoking still accounts for more life years lost than any other modifiable risk factor (Reitsma et al., 2017). The NHS cost in England of treating smoking-related illness is estimated at £2.6bn a year (Public Health England, 2017). ED attendance represents a powerful 'teachable moment' to consider long term health changes (Boudreaux et al., 2005). This is particularly important if the presenting condition could be seen as smoking related, for example a fracture due to bone weakening from smoking. For those attending hospital for other health reasons, stopping smoking is also very likely to improve outcomes related to their presenting condition (e.g. improved recovery times from infections or wound healing), which may act as a powerful motivator.

There is inconsistent evidence for smoking cessation interventions delivered in the ED (Lemhoefer et al., 2017), no UK evidence, and no studies have explored using e-cigarettes for smoking cessation support within the ED. E-cigarettes are now the most popular support method for smokers trying to stop (McNeill et al., 2018) and there is increasing evidence that they are an effective way to quit (Hajek et al., 2019). However initial start-up costs of approximately £25 and lack of information may be barriers to first use (English et al., 2018). Providing an e-cigarette starter kit and brief advice may

DocuSign Envelope ID: 77E6F4CA-8F41-42B4-8991-9A76FB043639

be a highly effective way of helping people to stop smoking, but has so far not been tested in populations of 'unmotivated quitters' attending an ED.

Initial economic modelling using data from a previous trial of smoking cessation in ED (Bernstein et al., 2015) using a high cost estimate for the intervention (£263 per person approached) and a low quit rate (4% compared to control) resulted in a cost per Quality Adjusted Life Year (QALY) of less than £4000. If the difference in quit rate is 7.3% then the intervention is calculated to be cost saving.

Existing evidence: We are not aware of any published or ongoing studies on a) brief interventions for smoking cessation in the UK in ED or b) e-cigarettes for smoking cessation in ED. A systematic review and meta-analysis of randomised controlled trials (RCTs) on ED-initiated smoking cessation interventions identified 11 studies, none conducted in the UK. The review found interventions can be effective at increasing point prevalence abstinence (RR 1.40, P=0.02) up to 12 months after the intervention (Lemhoefer et al., 2017). Included studies were rated moderate or weak in quality and only 4 of the 11 studies used biochemical confirmation and 6 had 3 month or less of follow up. Since the publication of this review, a further RCT evaluating an "ask, advise and refer" (to community smoking cessation services) approach in ED found that 30 day quit rates were higher in the intervention group at 12 months, but the difference was not statistically significant (Cheung et al., 2018). There is good research evidence for emergency department-based alcohol brief intervention (Nilsen et al., 2008), screening for domestic violence (Ramsay et al., 2002), brief intervention for suicide prevention (Stanley & Brown 2012), and promoting child safety (Shields et al., 2013). None of these have been widely implemented within UK EDs (Bensberg & Kennedy 2002) due to the time pressures on staff and the focus on the treatment of acute issues, this trial addresses this issue by having a member of staff dedicated to delivering the smoking cessation intervention. This approach has been shown to be effective within mental health services (Robson et al., 2013).

A Cochrane review identified four RCTs suggesting e-cigarettes are more effective than placebo (Hartmann-Boyce et al., 2021). An RCT comparing free (no cost) cartridge e-cigarettes with low nicotine delivery alone, compared with nicotine patches, suggested a similar low efficacy for both treatments (Halpern et al., 2018). A UK RCT comparing a group assigned to an e-cigarette starter pack and a group assigned to nicotine replacement therapy (NRT) of their choice, both with associated behavioural support, found significantly higher (18.8%) biochemically verified one year smoking abstinence in the e-cigarette group compared with 9.9% in the NRT group (Hajek et al., 2019). This finding has since been replicated internationally, in a pragmatic, three-arm, parallel-group trial in New Zealand, finding that participants using NRT patches plus nicotine e-cigarettes had significantly higher CO-verified continuous abstinence at 6 months compared with those using patches plus nicotine-free e-cigarettes (Walker et al., 2020). Both the Hajek and Walker trials recruited populations of motivated quitters.

Survey and qualitative studies support trial evidence but additionally suggest that e-cigarettes as an intervention for smoking cessation may be particularly useful for populations of unmotivated quitters. Potentially, e-cigarettes encourage smokers to switch to vaping as a preferred option over smoking (Notley et al., 2018). Pilot data informing an ongoing feasibility trial has suggested that e-cigarettes may be beneficial as a harm reduction measure for vulnerable populations such as those who are homeless (Dawkins et al., 2019), and e-cigarettes are now widely accepted as a smoking cessation or temporary smoking abstinence tool within inpatient mental health settings and prisons

DocuSign Envelope ID: 77E6F4CA-8F41-42B4-8991-9A76FB043639

(ASH 2019a). However, as many current smokers will have at least tried an e-cigarette (Smoking In England, 2019) at some point, it is clear that access to a quality product, and brief advice on how to use the e-cigarette optimally to support smoking cessation, is required, particularly for vulnerable populations. Population level data show a trend in increased use of e-cigarettes (ASH 2019b) against a backdrop of falling tobacco smoking prevalence (Office for National Statistics 2018b), potentially linking the use of e-cigarettes and smoking cessation.

A Public Health England evidence review suggested e-cigarettes are 95% less harmful to health than smoking tobacco (McNeill et al. 2018; McNeill et al. 2021) and this estimate was supported by a Royal College of Physicians report (Royal College of Physicians, 2016). In view of the uncertainty around long term safety, traditional methods of smoking cessation are preferable to e-cigarettes (Bullen et al., 2013), but for those who have tried and failed to quit smoking, switching to e-cigarettes is recommended as a harm reduction option. Studies have demonstrated decreased exposure to carcinogens for smokers switching completely from tobacco to e-cigarettes. Whilst some exposures were detected, levels were comparable to those who quit smoking using prescribed nicotine replacement therapy (Shahab et al., 2017).

Recent reports from the USA highlight a small number of deaths associated with vaping and a cluster of serious respiratory conditions. However, these cases have almost certainly been caused by vaping vitamin E acetate which is used as a dilutant in tetrahydrocannabinol oils (Blount et al., 2019) or contaminated e-liquids in the US market where regulations are less stringent than in the UK (Beaglehole et al., 2019). A recent study also found that within one month of quitting smoking, participants who switched to e-cigarettes (with or without nicotine) had a significant improvement in vascular function (George et al., 2019).

4.1.1 Explanation for choice of comparators

This research will provide a much-needed evidence base as to whether a smoking cessation intervention delivered in EDs can increase smoking cessation rates. This is key in meeting Government targets to continue to reduce smoking prevalence and target those in greatest need, including those in lower socioeconomic and hard to reach groups, reducing health inequalities (Department of Health, 2017). A secondary element is the effectiveness of e-cigarettes for smoking cessation in smokers not planning a quit attempt.

4.2 Objectives

Aim:
To undertake a randomised controlled trial (RCT), with internal pilot, comparing a brief intervention (including provision of an e-cigarette) with signposting to smoking cessation services, assessing long term smoking abstinence in people attending an Emergency Department.

Objectives:

1. To run an internal pilot, with clear stop/go criteria, primarily to test recruitment systems.

2. To definitively test real-world effectiveness of an ED based smoking cessation intervention in comparison with usual care, by comparing smoking abstinence at 6 month follow-up between trial groups.

3. To undertake a cost effectiveness analysis of the intervention in comparison with usual care from an NHS and personal social services (PSS) perspective.

DocuSign Envelope ID: 77E6F4CA-8F41-42B4-8991-9A76FB043639

4. To undertake an embedded mixed-methods process evaluation to assess delivery, implementation, fidelity and contamination.

4.3 Trial Design

A two-group, multi-centre, pragmatic, individually randomised, controlled trial with an internal pilot, and including economic evaluation and process evaluation.

5 Methods

5.1 Site Selection

The trial sponsor has overall responsibility for site and investigator selection and has delegated this role to the CIs and NCTU.

5.1.1 Study Setting

The setting is five NHS hospital Emergency Departments preselected to maximise generalisability of findings based on being representative in deprivation, ethnicity, hospital size (including teaching hospital and district general hospital) and location of the variety of Emergency Departments within the UK. If there are issues with recruitment Addenbrooke's Hospital have agreed to assist by recruiting patients through their well-established ED NIHR network nurses.

5.1.2 Site/Investigator Eligibility Criteria

Once a site has been assessed as being suitable to participate in the trial, the trial team will provide them with a copy of this protocol.

To participate in the CoSTED trial, investigators and trial sites must fulfil a set of criteria that have been agreed by the CoSTED Trial Management Group (TMG) and that are defined below.

Eligibility criteria:

- A named academic or clinician is willing and appropriately qualified to take Principal Investigator responsibility
- Suitably trained staff are available to identify and recruit participants and enter data

Trial sites meeting eligibility criteria will be issued with the CoSTED Investigator Site File (ISF) and a pack of documentation needed by the Research and Development Department (R&D) of their Trust to enable the Trust to provide confirmation of capacity and capability to undertake the study.

The five sites for this study have been preselected on the basis of existing collaborations, ongoing engagement with the trial team, and members of the trial team already identified as lead for each site. The following sites will be included the:

1. Norfolk and Norwich University Hospital (NNUH)
2. Royal London Hospital (RLH)
3. Homerton University Hospital (HUH)
4. Leicester Royal Infirmary (LRI)
5. Royal Infirmary of Edinburgh (RIE)
6. Addenbrooke's Hospital (ADH)

DocuSign Envelope ID: 77E6F4CA-8F41-42B4-8991-9A76FB043639

5.1.2.1 Principal Investigator's (PI) Qualifications and Agreements

The investigator(s) must be willing to sign an investigator statement to comply with the trial protocol (confirming their specific roles and responsibilities relating to the trial, and that their site is willing and able to comply with the requirements of the trial). This includes confirmation of appropriate qualifications, agreement to comply with the principles of GCP, to permit monitoring and audit as necessary at the site, and to maintain documented evidence of all staff at the site who have been delegated significant trial related duties.

5.1.2.2 Resourcing at site

The investigator(s) should be able to demonstrate a potential for recruiting the required number of suitable subjects within the agreed recruitment period. They should also have an adequate number of qualified staff and facilities available for the foreseen duration of the trial to enable them to conduct the trial properly and safely.

Sites will be expected to complete a delegation of responsibilities log and provide staff contact details.

The site should have sufficient data management resources to allow prompt data return to NCTU.

5.2 Site approval and activation

On receipt of the signed investigator statement, site agreement, approved delegation of responsibilities log and staff contact details, written confirmation will be sent to the site PI. The trial manager or delegate will notify the PI in writing of the plans for site initiation. Sites will not be permitted to recruit any patients until a letter for activation has been issued. The Trial Manager or delegate will be responsible for issuing this after a green light to recruit process has been completed.

The site must conduct the trial in compliance with the protocol as agreed by the Sponsor, Health Research Authority (HRA), and which was given favourable opinion by the Research Ethics Committee (REC). The PI or delegate must document and explain any deviation from the approved protocol and communicate this to the trial team at NCTU.

5.3 Participants

5.3.1 Eligibility Criteria

There will be **NO EXCEPTIONS** (waivers) to eligibility requirements at the time of randomisation. Questions about eligibility criteria should be addressed **PRIOR** to attempting to randomise the participant.

The eligibility criteria for this trial have been carefully considered and are the standards used to ensure that only medically appropriate participants are entered. Participants not meeting the criteria should not be entered into the trial for their safety and to ensure that the trial results can be appropriately used to make future treatment decisions for the behaviour of smoking. It is therefore vital that exceptions are not made to these eligibility criteria.

Participants will be considered eligible for enrolment in this trial if they fulfil all the inclusion criteria and none of the exclusion criteria as defined below. For those requiring translation, the method already used for the triage and clinical appointment in the ED will be used, following local policies.

DocuSign Envelope ID: 77E6F4CA-8F41-42B4-8991-9A76FB043639

This could include formal in-person or telephone translation (if available) by a professional translator, if feasible and the participant consents. Only if there is no possibility of translation being available, will the potential participant be excluded.

5.3.1.1 Participant Inclusion Criteria

1. Adult ≥ 18 years old.
2. Current daily tobacco smoker (self-reporting smoking of at least one cigarette, or equivalent, per day).
3. Attending the ED for medical treatment (or accompanying someone attending for medical treatment).
4. Submitting an expired carbon monoxide (CO) breath test reading of more than ≥8 ppm.

5.3.1.2 Participant Exclusion Criteria

1. Requiring immediate medical treatment as defined by the treating clinician (on ethical grounds), therefore unavailable.
2. In police custody (due to difficulty providing the e-cigarette if randomised to intervention)
3. A known history of allergy to nicotine replacement products.
4. Currently defined as a dual user (already using an e-cigarette daily as well as smoking tobacco daily).
5. Without the capacity to give informed consent for participation in the study.
6. Have taken part in the CoSTED trial already.

5.3.1.3 Procedure for accompanying person

If the person accompanying a presenting patient is also a smoker is eligible to participate in the study and provides consent to participate, they can be enrolled into the study and randomised along with the patient. The accompanying person can also be enrolled in the study and randomised even if the eligible patient refuses consent. Inclusion of an accompanying person will occur at the discretion of the site researcher, based upon capacity and opportunity. Details of the process for this are in 5.3.1.7 below.

5.3.1.4 Procedure if admission is required or participant requires immediate medical treatment

Should a participant be admitted to hospital they will not be excluded from the trial, and if allocated to the CoSTED intervention will still receive it in the emergency department, including provision of the e-cigarette starter kit. They will be given information of the local hospital smoking and e-cigarette policies.

Should a participant require immediate medical treatment on arrival at ED (within ED), they can still enter the trial if and when they then become available and able to be consented and randomised.

5.3.1.5 Eligibility Criteria for Individuals Performing the Interventions

Smoking cessation advisors' delivering the CoSTED intervention will be required to have been trained to deliver the CoSTED smoking cessation advice intervention using the intervention protocol, as evidenced in the intervention training record and site delegation log. They will have completed a post-training questionnaire to confirm completion and to evaluate the training.

Pope I, *et al*. *Emerg Med J* 2024; 41:276–282. doi: 10.1136/emermed-2023-213824

BMJ Publishing Group Limited (BMJ) disclaims all liability and responsibility arising from any reliance placed on this supplemental material which has been supplied by the author(s)

DocuSign Envelope ID: 77E6F4CA-8F41-42B4-8991-9A76FB043639

5.3.1.6 Co-enrolment Guidance

Co-enrolment will be permitted. However, co-enrolment requests in similar behavioural change interventions may be reviewed on a case-by-case basis in accordance with NIHR-supported co-enrolment guidelines (e.g. given COVID research priorities).

5.3.1.7 Screening Procedures and Pre-randomisation Investigations

Written (either on paper or electronically) informed consent to enter and be randomised into the trial must be obtained from participants after an explanation of the aims, methods, benefits and potential hazards of the trial and **BEFORE** any trial-specific procedures. The only procedures that may be performed in advance of written informed consent being obtained are those that would be performed on all patients in the same situation as a usual standard of care. Through this, sites will be required to collect a CO breath test reading once consent has been obtained, but before randomisation, to confirm eligibility.

Potentially eligible participants will be identified at during their ED attendance by ED research, medical and/or nursing staff where it is locally agreed that they are part of the clinical care team. They will ask the smoking status of each patient and provide them with an information sheet about the trial if they report smoking tobacco. Potential participants will then be approached by a member of the site research team in the ED, who will explain the nature of the trial and, if willing, consent them, confirm eligibility (using carbon monoxide testing), ask them to complete the baseline questionnaires and randomise them.

In the event of the person accompanying the patient also meeting inclusion criteria they will be dealt with as follows: if the patient does not consent to participate but the person accompanying them does consent, the person accompanying the patient will be randomised as normal and included in the primary analysis and count towards the sample size. If both the patient and the person accompanying them consent to participate then the patient will be randomised and be included in the primary analysis and the accompanying person will receive the same treatment (intervention or control) but will NOT be included in the primary analysis, count towards the sample size, or be counted as a separate participant for site accruals. This decision will be recorded prior to randomisation to prevent introducing bias.

The Principal Investigator (PI) retains overall responsibility for the informed consent of participants at their site and will ensure that any person delegated responsibility to participate in the informed consent process is duly authorised, trained and competent to participate according to the ethically approved protocol, principles of Good Clinical Practice (GCP) and Declaration of Helsinki.

Eligible patients who are approached but who do not wish to participate will be anonymously recorded as part of a screening log, researchers will capture information on reason(s) for declining participation where possible (this information is not required for accompanying people).

5.4 Internal Pilot

The Independent Data Monitoring Committee (DMC) and independent Trial Steering Committee (TSC) will scrutinise recruitment and protocol fidelity at 3 months into recruitment to establish continuation or stopping the trial at the pilot stage. Information based on the below red/amber/ green recruitment targets will be provided to the committees for scrutiny.

DocuSign Envelope ID: 77E6F4CA-8F41-42B4-8991-9A76FB043639

Green: If, 3 Months after the start of recruitment **any** of the following criteria are met or exceeded then we expect the trial to recruit >972 (90% power) within the 12 months period.

1. Average monthly recruitment per site in Month 3 = 19
2. Monthly trial recruitment in Month 3 = 95
3. Cumulative randomisations by end of month 3 = 159

Amber: If 3 Months after the start of recruitment the **green** criteria above have not been met but the **amber** criteria below have been met then then the trial will be on course to only recruit to approximately 712 (80% power) within the 12 months period and would require approximately a 3 month extension to recruitment to reach 90% power.

1. Average monthly recruitment per site in Month 3 = 15
2. Monthly trial recruitment in Month 3 = 75
3. Cumulative randomisations by end of month 3 = 125

If recruitment levels are between amber and green, then an urgent TSC meeting will be convened to discuss strategies to improve site / participant recruitment and the possibility of opening additional sites. If improvements do not occur by Month 6 then the funder will be notified and the TSC will meet again to discuss whether any other changes to the project can be made or whether consideration of discontinuing the study should be made.

Red: If 3 Months after the start of recruitment none of the **amber** criteria above are met then it is unlikely that the trial will recruit enough participants for 80% power, even if a short extension to recruitment was provided. If this is the case the TSC will meet to discuss whether any other changes to the project can be made or whether consideration of discontinuing the study should be made.

5.5 Interventions

There are two trial arms:

- Arm A – Treatment as usual (TAU)
- Arm B – CoSTED Intervention

5.5.1 Arm A – Treatment as Usual (TAU)

All trial participants will be offered signposting to NHS smoking cessation services through provision of written information about local services. This information will be given to the participant by the member of the research team taking consent, after randomisation.

5.5.2 Arm B – CoSTED Intervention

In addition to TAU, those randomised to the intervention arm will receive the CoSTED intervention. The CoSTED intervention is an opportunistic smoking cessation intervention comprising three elements: 1) brief smoking cessation advice, 2) the provision of an e-cigarette and training in its use and 3) referral to local stop-smoking services.

5.5.2.1 Intervention components

1. <u>Brief smoking cessation advice (up to 15 minutes)</u>

The brief smoking cessation advice will follow a protocol, last no more than 15 minutes, and be undertaken in the Emergency Department. The trial team will be flexible about space in which to

DocuSign Envelope ID: 77E6F4CA-8F41-42B4-8991-9A76FB043639

deliver the intervention in order to deal with variability in demand for physical space within the ED. Following trial specific intervention training, the advice will be delivered by a smoking cessation advisor. Protocol driven advice will address key aspects of the importance of quitting smoking, tailored to the participant where possible (e.g. discussing improved wound healing for patients attending with an open wound, or discussing increased risk of fractures for those who continue to smoke, for those attending with a fracture). This part of the intervention is a single 15-minute session undertaken within the ED department, in a quiet area or in a separate room if available.

2. <u>The provision of an e-cigarette starter kit and training in its use (up to 15 minutes)</u>

An e-cigarette starter kit which includes about a week of liquid and training on its use lasting up to 15 minutes will be provided including how to use an e-cigarette efficiently to satisfy nicotine cravings (e-cigarette device specific 'training').

The choice of which e-cigarette to provide will be based on available devices and feedback from Patient and Public Involvement (PPI). It will be one with a closed eliquid cartridge for ease of use, produced by an independent company not funded by the tobacco industry. A limited choice of e-liquid flavours will be offered in line with current UK regulations (e.g. a fruit, a menthol and a tobacco flavour), with a high strength nicotine liquid (typically 16-20mg nicotine per millilitre), as higher nicotine strength is indicated for heavier smokers, and modern devices with improved nicotine delivery may be more clearly linked to cessation (Hartmann-Boyce et al., 2021). Information about where to purchase ongoing cartridges will be provided. E-cigarette safe-use and charging information is provided in the leaflet enclosed within the starter-pack.

3. <u>Referral to stop-smoking services (included within the time above)</u>

Participants randomised to the intervention group will also be offered a referral to the local stop smoking service who will provide routine follow up. Agreement has been gained from smoking cessation services to support the trial. The services are happy to receive referrals from the trial and provide their standard follow up services (including face to face or telephone support) and provide the trial team with referral and engagement data.

The smoking cessation advisor will complete a brief feedback form after each intervention contact to monitor timing of intervention delivery, fidelity and 'dose'.

5.5.2.2 Training for those delivering the intervention

Those delivering the intervention will undertake approximately 2.5 days of standardised training and will be provided with an intervention manual. Training will be delivered using both online e-learning and live online courses, some of which will be modular, comprising:

1. Generic remote training package (approx. 1.5 days in total):
- Core modules (overview) and speciality modules (E-cigarettes, mental health, pregnancy and smoking cessation, and second-hand smoke) from the National Centre for Smoking Cessation Training and Assessment Programme (modular, completed in own time).
- Smoking Cessation Advisor - Level 2 Training, delivered by *Smoke Free Norfolk* (approximately 4 hours) online or face-to-face.

2. Bespoke Training, delivered online or face-to-face by the CoSTED Team in liaison with *Smoke Free Norfolk* (1 day), to include:
- CoSTED Induction – introduction to RCTs, and background to CoSTED, understanding administration of the trial and intervention
- Introduction to smoking and e-cigarettes, including up-to date e-cigarette research
- Smoking impacts on common health conditions presenting at ED
- Brief smoking cessation advice training
- E-cigarette provision and demonstration training

DocuSign Envelope ID: 77E6F4CA-8F41-42B4-8991-9A76FB043639

5.5.3 Accountability

The site team will be responsible for providing the e-cigarette starter kits to participants allocated to the intervention arm and will indicate on the database this has been issued. Database checks will be built in to notify NCTU operations and relevant site staff of each participant allocated to the intervention arm. Confirmation of receipt of 1) the e-cigarette starter kit, 2) information about where they can purchase ongoing supplies, and 3) a package of information resources, will be collected by the smoking cessation advisor from each participant and added to the database for the audit trail. Participants will not be required to return the e-cigarette at the end of the study.

5.5.4 Compliance and Adherence

The study may be monitored or audited in accordance with the current approved protocol, principles of GCP, relevant regulations and Standard Operating Procedures (SOPs). A rigorous quality control programme will be adopted to ensure protocol and intervention fidelity.

Our methods to enhance and assess intervention fidelity are informed by the NIH Behaviour Change Consortium fidelity framework (Bellg et al., 2004).

1. Delivery and quality of training: We will evaluate the training of all smoking cessation advisors that are trained to deliver the intervention, in online meetings to troubleshoot any issues, and evaluate the training through a post-training questionnaire.
2. Delivery, quality, and consistency of intervention delivery by smoking cessation advisors: We will discuss intervention fidelity during the training, introducing core components that need to be delivered and how delivery can be adapted to the participant and their context, followed by discussion. We will have regular online meetings of the intervention team (all smoking cessation advisors and research team members involved in intervention delivery) to share good practice and problem-solve any challenges including fidelity of delivery. All intervention smoking cessation advisors will receive an intervention manual to support fidelity of intervention delivery. Some of the sessions will be observed, with those to be observed being determined centrally and communicated to the advisor.
3. Participant engagement and enactment (participant fidelity as part of our process evaluation). This will be assessed quantitatively through monitoring of responses to questions at 6 months. Qualitatively we will assess fidelity through our interviews with a sub-sample of participants on completion of the study. This fidelity data will be used to illuminate the trial findings.

The site PI and other key staff from each centre will attend online or face-to-face site initiation training, coordinated by the trial team. This will incorporate elements of trial-specific training necessary to fulfil the requirements of the protocol. This will occur before site activation is given. This site initiation training visit will consist of reviewing the trial protocol, recruitment, consent, randomisation, follow-up, trial procedures, intervention training as applicable, trial arrangements, data protection and data handling.

All training undertaken by site researchers will be recorded on a training log and the roles of each member recorded on a site signature list and delegation of responsibilities log. The site PI will be responsible for ensuring that this accurately reflects the training undertaken and roles and responsibilities of each site researcher. This will be stored in each site's Investigator Site File (ISF) and up to date copies returned to the central trial office after each update.

DocuSign Envelope ID: 77E6F4CA-8F41-42B4-8991-9A76FB043639

5.5.5 Crossover between trial groups

Those randomised to the control group will not receive the CoSTED intervention, and only those randomised to the experimental intervention group will receive the CoSTED intervention. It is, however, possible that control participants could, independent of the trial, purchase their own e-cigarettes and/or approach local smoking cessation services themselves. As this is a real-world trial, we will not attempt to control for this but will ask about e-cigarette use in each group in the CRF at 6 month follow up.

5.5.6 Concomitant Care

All participants will receive treatment they attend ED for, regardless of randomisation into this trial. Respecting the pragmatic nature of this trial, participants will not be asked to desist from receiving other forms of treatment during this trial such as general practitioner (GP) consultations, medication changes or alternative treatments if required. Use of these treatments will be recorded through a self-reported service use questionnaire at the 6 month follow-up point.

5.5.7 Protocol Intervention Discontinuation

In consenting to the trial, participants are consenting to engaging with the trial intervention, trial follow-up and data collection. However, an individual participant may discontinue intervention for any of the following reasons:

- Unacceptable intervention toxicity or adverse event
- Inter-current illness that prevents further intervention
- Any change in the participant's condition that in the clinician's opinion justifies the discontinuation of the intervention
- Withdrawal of consent for intervention by the participant

As participation in the trial is entirely voluntary, the participant may choose to discontinue trial intervention at any time without penalty or loss of benefits to which they would otherwise be entitled. Although not obliged to give a reason for discontinuing their trial intervention, a reasonable effort should be made to establish this reason, whilst remaining fully respectful of the participant's rights.

Participants who discontinue protocol intervention, for any of the above reasons, should remain in the trial for the purpose of follow up and data analysis unless they wish to also discontinue providing data. In this case, data up to the point of discontinuation will be retained for the purposes of analysis but no further data collected. NCTU should be informed of the intervention discontinuation in writing using the appropriate CoSTED trial documentation.

5.6 Outcomes

5.6.1 Primary Outcomes

The primary effectiveness outcome will be self-reported continuous smoking abstinence, biochemically validated by CO monitoring at 6 months with cut off of ≥8ppm (i.e. a reading of 7 or less will denote abstinence), according to the Russell standard (SRNT 2002, West et al., 2005; Raiff et al., 2010). Participants who self-report having quit smoking at 6 months will then have their report verified by a carbon monoxide (CO) breath test, undertaken, by a researcher at the hospital where they were recruited or at their home, or by remote CO testing, to confirm cessation.

DocuSign Envelope ID: 77E6F4CA-8F41-42B4-8991-9A76FB043639

5.6.2 Secondary Outcomes

Secondary outcomes measured at 6 months from randomisation:

1. 7-day point prevalence abstinence (i.e. current smoking status, self-report of having smoked no cigarettes (not even a puff) in the past seven days, biochemically validated by carbon monoxide monitoring with cut off of ≥8 ppm)
2. Number of quit attempts
3. Time to relapse (if applicable)
4. number of cigarettes per day
5. Nicotine dependence (Heatherton et al. 1991)
6. Number of times using an e-cigarette per day
7. Incidence of self-reported dry cough in past week (yes/no) (Hajek et al, 2019)
8. Incidence of mouth or throat irritation
9. Motivation to Stop Smoking (Kotz et al, 2013) (single question)
10. Self-reported use of healthcare services in the last 6 months
11. Self-reported use of smoking cessation services in last 6 months
12. Quality of life (using the EQ-5D-5L) (EuroQol, 2019) and

Self-reported smoking status and adverse events/reactions will be collected at 1 and 3 months.

Given PPI feedback, 1 and 3 month follow-ups will initially be via text message and then via phone call, e-mail or post as required if there is no response to the initial text message. This will be conducted centrally by the core research team.

5.6.3 Process Evaluation

Participant interviews

The objective of this mixed methods process evaluation (Moore et al., 2015) is to assess implementation and explore participant views on the intervention compared to usual care, contextual variation and potential contamination between the intervention and control group. Detailed qualitative data will be collected through semi-structured interviews with a purposive sample of both intervention and control group participants (total n=30) after completion of final follow up, triangulated with brief observations of ED settings undertaken by the research team. Participants will have been given the option at baseline to consent to being contacted about the qualitative interviews.

A purposive sample will be selected from those who have agreed to be contacted by a member of the research team for the interview. Targeted demographics for the sampling frame will include: age, ethnicity, current smoking status and socioeconomic status. Those approached for interview will be contacted by email or telephone by a researcher and given detailed information on what is involved. They will be provided with a Participant Information Sheet (PIS) specifically for the qualitative interviews. After reading the PIS, participants can confirm their consent either by completing and returning a paper consent form or completing an electronic Informed Consent Form, accessed via a link in an e-mail or text. Interviews will be undertaken face-to-face or remotely via telephone or video-conferencing (e.g. Microsoft Teams) and audio-recorded for transcription. The topic guide will enquire about views and experiences of the intervention, barriers and facilitators, and explore the patient's longer-term perspective beyond the intervention period.

DocuSign Envelope ID: 77E6F4CA-8F41-42B4-8991-9A76FB043639

Thirty face-to-face or telephone/video call interviews will be conducted, split equally across the trial arms and across the five sites. Interviews will be conducted up to four weeks after the 6 month follow-up has been completed. Interviews will be semi-structured, following an open-ended question schedule, and last around 45 minutes. Interview guides, developed in consultation with PPI representatives will capture views and experiences of the intervention to understand barriers and facilitators, and to assess patient perspectives. We will explore continued use of an e-cigarette to understand experiences beyond the initial intervention that may impact on long term smoking abstinence outcomes. Emerging themes from the qualitative interviews will be discussed with PPI members.

Smoking cessation advisor interviews

At least one smoking cessation advisor from each of the 5 ED sites delivering the intervention will also be invited to take part in a qualitative interview on completion of recruitment. Staff interviews will assess views and experiences of intervention delivery, giving an insider perspective on which parts of the intervention package are deemed to be most helpful, in which circumstances, with which participants. Barriers and facilitators to intervention delivery will be explored from the staff perspective, to aid interpretation of trial findings and triangulate with participant qualitative data.

Site Observations

Observational data of the intervention delivery and ED setting will be collected at each of the five ED sites. Each observation visit will be pre-arranged with the staff based at each site and will occur during usual working hours. Consent for the participant intervention observations will be obtained in the baseline consent. A researcher will attend for around three hours and take notes about the context, the interactions and conversation between patients approached for the trial and staff involved. A structured observation record sheet will be used for this purpose, prompting the researcher to observe the environment, interactions between key actors, the culture of the department, the flow of patients and any critical events that may impact on intervention delivery and implementation. The researcher will also broadly document details of the setting, including any smoking and vaping observed in and around the department. This will aid in assessing context of intervention delivery. A Site Profile Form, completed prior to site observations as part of the Site Initiation Visit (SIV), will capture information that will support the researcher observations.

5.6.3.1 Managing distress or harm following participant interviews

Interviews will occur after completion of participation in the trial at 6 months. A participant can pause or stop the interview at any time they wish. If any participant were to become distressed during the interviews, the researcher, as a duty of care, would provide verbal support and time for that individual to discuss their distress to try to support them. In the instance that they remained concerned regarding the welfare of that individual, they may recommend the individual contact their general practitioner for further support and advice. In all instance, this will be recorded within the qualitative research notes.

DocuSign Envelope ID: 77E6F4CA-8F41-42B4-8991-9A76FB043639

5.7 Participant Timeline

5.7.1 Table 1: Schedule of enrolment, interventions and assessments

	**Screening**	**Eligibility/ Baseline**	**Smoking advice**	**1 month follow up**	**3 month follow up**	**6 month follow up**
**Type of contact**	**Member of ED clinical care team**	**Member of the site research team**	**Site smoking cessation advisor**	**Text message**		**Link to online CRF or paper sent**
Eligibility	X					
Participant Information Sheet	X					
Informed Consent		X				
Check eligibility including CO monitor reading		X				X
Demographics		X				
Self-reported smoking status		X		X	X	X
Quit attempts		X				X
Time to relapse						X
Cigarette & tobacco use		X				X
Nicotine dependence (Heatherton et al. 1991)		X				X
Motivation to stop smoking (Kotz et al. 2013)		X				X
E-cigarette use		X				X
Incidence of dry cough		X				X
Incidence of mouth or throat irritation		X				X
Quality of life - EQ-5D-5L (EuroQol 2018)		X				X
Self-reported use of healthcare services		X				X
Adverse events				X	X	X
Self-reported use of smoking cessation services		X				X
Randomisation		X				
Smoking Cessation Intervention			X			
Referral to smoking cessation service			X			
E-cigarette given			X			
Signposting to NHS smoking cessation services			X			

Shading indicates only undertaken by those in the intervention group

DocuSign Envelope ID: 77E6F4CA-8F41-42B4-8991-9A76FB043639

5.7.2 Patient Assessments

5.7.2.1 Screening

Informed consent to enter and be randomised into the trial must be obtained from participants after explanation of the aims, methods, benefits and potential hazards of the trial and before any trial-specific procedures. The only procedures that may be performed in advance of written informed consent being obtained are those that would be performed on all patients in the same situation as a usual standard of care.

Members of the research team at each site will engage with clinical ED team on how best to identify participants. During this screening process potential participants will be asked by a member of the clinical team (e.g. triage nurse) about their smoking status to determine eligibility for the trial.

The clinical team member will ask for verbal consent from potentially eligible patients to transfer their name to a member of the site research team. They will provide patients with a study participant information sheet (PIS), and be given the opportunity to read the PIS and make a decision on whether they, and/or the person/people accompanying them think they are eligible and would like to take part in the research.

Once the potential patients have read the PIS, had the opportunity to ask a member of the site research team any questions, and completed the paper/electronic informed consent form, a member of the site research team will check eligibility which includes providing a CO reading (which will need to be ≥8ppm for inclusion).

5.7.2.2 Baseline

Once the participant has given informed consent and eligibility is confirmed, including taking a CO reading, the participant will be provided with the baseline questionnaire to complete on a tablet or paper. This will consist of demographics, reason for ED attendance, smoking status, nicotine product use, e-cigarette use, use of healthcare services in the past 6 months, use of smoking cessation services in the past 6 months, self-reported dry cough and throat irritation, the Fagerstrom test for nicotine dependence (FND), the Motivation to Stop Smoking (MTSS) question, and quality of life (EQ-5D-5L). The completion of the baseline measures will trigger the database randomisation, notifying the trial team and local research team of the patient’s trial arm.

5.7.2.3 Smoking cessation advice

Once randomised, those allocated to receive the intervention will be seen by a smoking cessation advisor for a smoking cessation brief intervention. The advice appointment will last for up to 30 mins and include smoking cessation advice (up to 15 mins), support in using an e-cigarette (up to 15 mins) and referral to local smoking cessation services.

5.7.2.4 1-month and 3 month Follow-Up

At one- and three-months post-randomisation, text messages will be automatically generated by the study database and sent to participants in both arms of the trial to assess smoking status and any hospital attendances (to capture potential adverse events). Participants will be asked to respond; if after two attempts, there is no response, a member of the central trial team will follow-up by email

DocuSign Envelope ID: 77E6F4CA-8F41-42B4-8991-9A76FB043639

or telephone. For those that do not have a mobile telephone, they will be telephoned on their home number or e-mailed to retrieve this information.

5.7.2.5 6 Month Follow-up

At 6 months post randomisation, a link will be automatically generated by the database and sent by SMS/email to participants in both arms of the trial to complete the 6 month follow up questionnaire. The questionnaire will consist of smoking status, time to relapse (if applicable), nicotine product use, e-cigarette use, use of healthcare services in the past 6 months, use of smoking cessation services in the past 6 months, self-reported dry cough and throat irritation, the Fagerstrom test for nicotine dependence (FND), the Motivation to Stop Smoking (MTSS) question, and quality of life (EQ-5D-5L) and health service use (which will capture potential adverse events). If participants do not respond to the link, they will be sent one follow-up reminder one week later. If the link is still not clicked a member of the central trial team will follow-up by email, telephone, or text. If participants are unable or unwilling to complete the 6 month questionnaire electronically, a member of the central trial team will contact participants by telephone or post to complete the 6 month questionnaire.

Carbon monoxide concentration will be measured in those who report smoking abstinence in order to verify their smoking status. There will be several options for this, to maximise data collection. They can either:
1) return to the hospital they attended at baseline (they will make an appointment with a member of the site research team),
2) undertake remote collection with a CO monitor that will be sent to them (an appointment will be made with the central team for a video-call to retrieve the reading and then they will return the monitor in a stamped addressed envelope), or
3) be visited at their home by a member of the local or central research team.

On completing the 6 month follow-up (including CO verification if required) the randomised participants will be reimbursed for their time with a £30 voucher.

Participants will be asked in their baseline consent if they consent to being contacted after completion of the trial to take part in a qualitative interview. If they agreed, this interview will take place after completion of the final questionnaire and submission of the final CO reading. Once completing the qualitative interview, the participants will be reimbursed for their time with a further £20 voucher.

5.7.3 Early Stopping of Follow-up

If a participant chooses to discontinue their trial intervention, they should continue to be followed up as closely as possible to the follow-up schedule defined in the protocol, providing they are willing. They should be encouraged and facilitated not to leave the whole trial, even though they no longer wish to take the trial intervention. If, however, the participant exercises the view that they no longer wish to be followed up, this view must be respected, and the participant withdrawn entirely from the trial. NCTU should be informed of the withdrawal in writing using the appropriate CoSTED trial documentation. Data already collected will be kept and included in analyses according to the intention-to-treat principle for all participants who stop follow up early.

DocuSign Envelope ID: 77E6F4CA-8F41-42B4-8991-9A76FB043639

Participants who stop trial follow-up early will not be replaced. Note that the primary outcome is assumed to be relapsed to smoking at 6 months on ITT if lost to follow up, although some sensitivity analyses will be conducted around this assumption.

5.7.4 Participant Transfers

As follow-up strategies are by text, e-mail and telephone, participant transfer from their consenting centre is not applicable.

5.7.5 Loss to Follow-up

A research team member will establish a rapport with participants and emphasise the importance of trial procedures and follow up. Contact by e-mail and / or text message and / or phone will be initiated prior to follow up points to maintain contact and maximise follow up. The trial team will be alerted by the database when participants are nearing their 6 month follow-up.

5.7.6 Trial Closure

The end of the trial is defined as 6 months following the last follow-up data collection point for the last patient randomised, to allow for data entry and data cleaning activities to be completed.

5.8 Sample Size

The sample size is based on an expected quit rate of 12.2% in the intervention group. This was used for the intervention rate based on a US trial of an Emergency Department smoking cessation intervention using a brief intervention, referral to smoking cessation services and nicotine replacement (Bernstein et al., 2015). A quit rate of 6.2% was used in the control group based on an average of 3 studies of unmotivated quitters who received either sign posting or no intervention (Hennrikus et al., 2010; McFall et al., 2010; Pieterse et al., 2001). This is broadly in keeping with the result of a recent Cochrane review of incentives which found a quit rate of 7.2% amongst controls (Notley et al., 2019). A sample size using 90% power and the above expected quit rates gives a required sample size of 972 (486 per group) at the 5% level of significance using a two-tailed test. No increase for drop-out has been made as dropouts, individuals for whom we do not have the primary outcome data, will be assumed to have returned to smoking. This assumption is standard in the smoking cessation literature as the pattern of missingness is not random (West et al., 2005).

5.9 Recruitment and Retention

5.9.1 Recruitment

Based on our detailed PPI observations at NNUH, RLH, HUH, RIE and LRI there were between 15 and 38 current smokers attending each ED between 8am and 4pm on weekdays. During PPI work it was found to be very feasible to identify at least 1 patient per hour at each centre who met inclusion criteria and would be willing to be recruited. To allow for challenges with identification and implementation the above timeline is based on recruiting 1 patient per weekday per site. Sites will generally be asked to recruit during usual working hours, but this will be flexible based on staff availability. Actual recruitment periods will be recorded by sites as part of the screening log. If there are issues with recruitment, Addenbrooke’s Hospital have additionally agreed to assist by recruiting patients through their well-established ED NIHR network nurses.

PPI work indicates that a direct approach whilst patients are waiting to see a clinician would be the most effective and most acceptable form of approach. Therefore, during the trial potential

DocuSign Envelope ID: 77E6F4CA-8F41-42B4-8991-9A76FB043639

participants will be identified at an early point in their ED attendance by being asked smoking status and being provided with an information sheet about the trial. Potential participants will then be approached by a member of the site research team located in the ED, who will explain the nature of the trial and, if willing, consent, check eligibility, support the completion of baseline questionnaires and randomise them. Randomisation will be undertaken by a third-party web-based system ensuring concealed allocation, developed and delivered by Norwich CTU.

Participants randomised to the intervention group will then receive a smoking cessation brief intervention, consisting of protocol driven brief tailored advice and advice on how to optimally use an e-cigarette for smoking cessation, delivered by a smoking cessation advisor plus the provision of an e-cigarette starter kit and training on its use.

The trial team will be flexible about space in which to deliver the intervention in order to deal with variability in demand within the Emergency Department. It is anticipated the brief intervention and e-cigarette training will take around 30 minutes in total. PPI indicates many current smokers are keen to try an e-cigarette but unsure where to start. Participants randomised to the intervention group will also be offered a referral to the local stop smoking service who will provide routine follow up, as PPI work indicates that ongoing support plays a key role in smoking cessation.

Participants who are randomised to the control group will receive usual care plus signposting to NHS smoking cessation services through provision of written information about local services.

5.9.2 Retention

To maximise retention and minimise loss to follow up, we will retain contact with study participants. There will be one text/email reminder sent if text questionnaires/forms are not completed by participants. If participants have not responded to the initial texts or reminders, then a member of the research team may contact up to 5 times to offer support. Patients will also be offered reimbursement for their time, (a £30 voucher), upon completing their 6 month follow-up.

5.10 Assignment of Intervention

5.10.1 Allocation

5.10.1.1 Sequence generation

Randomisation to treatment arm will take place after consent and baseline data has been collected, including CO verification of smoking status. The randomisation scheme will be computer generated by the NCTU data manager using a blocked design. Randomisation will be at the individual participant level (1:1) stratified by ED department.

5.10.1.2 Allocation concealment mechanism

The allocation is computer generated so will not be known prior to the participant being randomised. The participant will be allocated a participant number at time of consent. When the baseline data have been collected the research staff will then have access to the online randomisation process for that participant. The treatment allocation will be revealed and linked to that participant number. Allocation is concealed prior to randomisation to prevent treatment bias.

DocuSign Envelope ID: 77E6F4CA-8F41-42B4-8991-9A76FB043639

5.10.1.3 Allocation Implementation

The site trial team will be notified immediately by email of the participant allocation. They will notify the site research team and local smoking cessation advisor located within the ED and so available to the point of randomisation. For patients randomised to the CoSTED intervention, the local smoking cessation advisor trained in the intervention will then deliver the intervention in an appropriate area. Those randomised to the control group will be provided with signposting to NHS smoking cessation services through provision of written information (given to them by a member of the site research team) about local services, and remain in the ED to await review by a clinician.

5.10.2 Blinding

Due to the participatory nature of the intervention, it will not be possible to blind patients or treating advisors to group allocation. Members of the trial management team will not be blinded to the group allocation when looking at the summary statistics to ensure continuous comparison of intervention processes can be ascertained throughout the trial's progress.

5.11 Data Collection, Management and Analysis

5.11.1 Data Collection Methods

5.11.1.1 Trial Database

Source data worksheets will be drafted by the data manager with the CIs, Trial Managers, Trial Statistician and Trial Health Economist. These will be piloted and finalised. The database specification will be prepared by the NCTU data programmer and approved by the CIs, Trial Statistician and Trial Health Economist prior to the database being built. The database will be prepared by the NCTU Data Programmer and tested by the Trial Statistician, Trial Manager and trial site staff for user acceptability prior to the final system being launched.

Data collection, data entry and queries raised by a member of the CoSTED NCTU trial team will be conducted in line with NCTU and trial specific Data Management SOPs.

5.11.1.2 Data collection at site

Each participant will be given a unique trial Participant Identification Number (PIN). Clinical trial team members will receive trial protocol training. All data will be handled in accordance with the Data Protection Act 2018. Data will be collected at the time-points indicated in the Trial Diagram (see page 13).

Data will be collected by the site research teams (baseline collection), through text message at 1 and 3 months, and through a text/e-mail link to an online form (or on paper if requested) for the 6 month follow-up. Baseline data collection, including the service use questions, will be via direct online entry of data onto the central database by participants and/or members of the CoSTED trial team working within each research site. Data may be entered onto paper Case Record Forms (CRFs) prior to entry onto the database (but this is not an essential step). Staff will receive training on data collection and use of the online database system. Any paper CRF should be retained at site.

Paper identification logs, screening logs and enrolment logs will be kept at the trial site in a locked cabinet within a secured room. Staff will receive guidance on data collection and use of the online

DocuSign Envelope ID: 77E6F4CA-8F41-42B4-8991-9A76FB043639

system (see Section 5.2). Participant identifiable data will be stored in the database to enable patients to be contacted by site staff for the purpose of contacting participants and sending questionnaires; and the central Trial Office (NCTU) for the purpose for sending newsletters during the trial. There will be a clear logical separation of participant identifiable data from the trial data, in that role-based permissions will be configured such that users cannot access both the identifiable data and the trial data. All identifying fields will be tagged as such within the database, and permissions further configured to prevent these fields from being exported when trial data is extracted by members of the trial team.

A proportion of the intervention delivery at each site will be observed. A researcher will attend for around three hours and take handwritten notes about the context, the interactions and conversation between patients approached for the trial and staff involved. Immediately following each observation, notes will be typed up by the researcher who added their own thoughts and reflections.

Site smoking cessation advisors will complete post intervention forms following each intervention contact (a simple online data entry form onto the central database). This data will be used to monitor delivery and fidelity for the process evaluation.

At follow up a sub-sample of participants consenting to further contact will be contacted directly by the central research team to request separate consent to take part in a qualitative interview for the process evaluation. Detailed in 5.3.3 above.

Data collection, data entry and queries raised by a member of the CoSTED trial team will be conducted in line with the NCTU and trial specific Data Management Standard Operating Procedure.

5.11.2 Data Management

Data will be entered under the participants PIN number onto the central database stored on the servers based at NCTU, UEA. Access to the database will be via unique, individually assigned (i.e. not generic) usernames and passwords, and only accessible to members of the CoSTED trial team at NCTU, and external regulators if requested. The servers are protected by firewalls and are patched and maintained according to best practice. The physical location of the servers is protected physically and environmentally in accordance with University of East Anglia’s General Information Security Policy 3 (GISP3: Physical and environmental security).

The database and associated code have been developed by NCTU Data Management, in conjunction with the CoSTED trial team. The database software provides a number of features to help maintain data quality, including; maintaining an audit trail, allowing custom validations on all data, allowing users to raise data query requests, and search facilities to identify validation failure/ missing data.

After completion of the trial the database will be retained on the servers of NCTU for on-going analysis of secondary outcomes.

The identification, screening and enrolment logs, linking participant identifiable data to the pseudo-anonymised PIN, will be held locally by the trial site. This will either be held in written form in a locked filing cabinet or electronically in password protected form on hospital computers. After

DocuSign Envelope ID: 77E6F4CA-8F41-42B4-8991-9A76FB043639

completion of the trial the identification, screening and enrolment logs will be stored securely by the sites for 5 years unless otherwise advised by NCTU.

Qualitative interview data will be collected on an audio recorder, or by using computer recording via video conference, and immediately transferred to secure cloud storage (NCTU OneDrive) with access restricted to nominated members of the research team. Audio files will transcribed verbatim by a member of UEA administrative staff or by an external transcription company. Where possible, audio files will be auto-transcribed before being checked against the audio-recording. If using an external transcription service, a confidentiality agreement will be put in place and the recordings transferred via their encrypted software. When transcribed, the qualitative researcher will assign each transcript a unique study specific number and/or code and remove all identifying information from the transcripts. Only this anonymised form of data will be used for analysis and stored securely for 5 years unless otherwise advised by NCTU.

Observational notes will also be assigned a unique identifying code and stored securely at UEA with access restricted to nominated members of the research team.

5.11.3 Non-Adherence and Non-Retention

The consent form will explain that if a participant wishes to withdraw from the study, the data acquired prior to that point will be retained. Reason for withdrawal will be recorded, if given, as will loss to follow-up.

Non-adherence to intervention procedures will be assessed by the trained site team and recorded on the database by the site team.

5.11.4 Statistical Methods

See table 2 on page 39

5.11.4.2 Statistical Analysis Plan

A full statistical analysis plan will be reviewed and signed off by the independent DMC and TSC prior to analysis.

Descriptive statistics will be used to present the baseline characteristics of the two study groups. Analysis of smoking status will be based on the intention-to-treat principle by analysing individuals according to the treatment they were allocated to regardless of compliance. However, individuals for whom we do not have the primary outcome data will be assumed to have returned to smoking. This assumption is standard in the smoking cessation literature as the pattern of missingness is not random (West et al., 2005).

Missing data patterns will be examined, and if appropriate, multiple imputation will be undertaken. A full statistical analysis plan will be written and pre-registered in a published trial protocol. The comparison of smoking rates will be based on a logistic regression model adjusting for stratification variables. A sensitivity analysis will adjust for factors known to predict relapse. Self-reported abstinence rates will also be compared using a logistic regression model. The number of cigarettes and e-cigarettes will be compared using regression models. Other outcomes will be compared using

DocuSign Envelope ID: 77E6F4CA-8F41-42B4-8991-9A76FB043639

5.11.4.1 Table 2 - Outcomes

Outcome	Effect Size	Primary analysis model	Sensitivity analysis	Missing data	Other comments
Smoking abstinence<br>7-day point prevalence abstinence	Relative risk and risk difference	Log-binomial regression adjusting for site	Log-binomial regression adjusting for factors stratified in the randomisation and factors pre-specified by TMG before database lock.	Missing data will be assumed to have relapsed	Binary yes/no at 6 months
Number of quit attempts	Reported as medians	Mann-Whitney test	None	Missing data will be excluded, but a sensitivity analysis using multiple imputation will be undertaken.	None
Time to relapse	Hazard ratio	Cox proportional hazard model	None	Missing data will be assumed to have relapsed at the date the participant was last observed, e.g. if last visit is 6 months they will be assumed to have relapsed at 6 months.	None
Number of cigarettes per day<br>Number of times using an e-cigarette per day<br>Nicotine dependence<br>Motivation to stop smoking	Mean difference	Linear regression model adjusting for site.	None, but it is likely that the data will not be normally distributed to a non-parametric test will also be undertaken.	Missing data will be excluded, but a sensitivity analysis using multiple imputation will be undertaken.	None
Incidence of mouth or throat irritation<br>Incidence of dry cough	Relative risk and risk difference	Log-binomial regression adjusting for site	Log-binomial regression adjusting for factors stratified in the randomisation and factors pre-specified by TMG before database lock.	Missing data will be excluded, but a sensitivity analysis using multiple imputation will be undertaken.	None
Adverse events	None	None, the adverse event will be tabulated per group, no formal comparison will be undertaken.	None	None	None

DocuSign Envelope ID: 77E6F4CA-8F41-42B4-8991-9A76FB043639

similar regression models. The analysis plan may include analysis suggested by the PPI data and process evaluation, such as subgroup analysis or mediation analysis. Any analysis will be pre-specified in the analysis plan before the analysis is undertaken. A secondary analysis will be undertaken including the accompanying persons treating them as being clustered with the patient.

5.11.4.3 Additional Analyses

No additional analysis is planned at the moment, but if any become necessary prior to the analysis this will be documented in the SAP.

5.11.4.4 Analysis Population

The analysis population will be the intention to treat analysis, which will treat all randomised participants according to the group that they were allocated to regardless of compliance. If any individuals are found to subsequently be falsely included in the study, they will be discussed at TMG meetings and could potentially form post-randomisation exclusions.

5.11.4.5 Missing Data

As is standard in smoking cessation trials, individuals with missing data on the primary outcome and cessation data, including verification of abstinence through CO monitoring, will be firstly assumed to have returned to smoking (West et al., 2005). Additional analysis using multiple imputation will also be conducted using chained equations.

5.11.5 Economic evaluations

Economic evaluation will take the form of an incremental cost-effectiveness analysis from an NHS and PSS perspective on an intention-to-treat basis following NICE guidance (NICE, 2013). The costs will comprise intervention/usual care costs and general health care service costs. Intervention costs will be prospectively recorded alongside the intervention delivery. These include costs of training smoking cessation advisors for intervention delivery, costs of providing e-cigarette starter kit, costs of advisor time delivering the brief advice and the costs of local smoking cessation services engagement afterwards. Usual care costs will be the printing costs of written information of local services available. A self-completion service use questionnaire will be used to collect participants' utilisation of selected health care services at baseline and 6 months follow-up, covering the 3 months before baseline and 6 months after baseline respectively. A set of national average unit costs extracted from secondary sources of the appropriate year (PSSRU 2018; NHS Improvement, 2019) will be applied to quantities to derive a health care cost profile. All costs will be presented in Pound Sterling and the price year will be matched to the trial conducting time. In addition, we will also collect participants' out-of-pocket spending in relation to smoking cessation to capture potential financial burdens from participants' side.

We will use two outcome measures for effectiveness. One is the primary outcome of the trial: smoking abstinence at 6 months post-randomisation which will be used in the cost effectiveness analysis. The other is Quality Adjusted Life Years (QALYs), derived from EQ-5D-5L, for the cost utility analysis. We will administer EQ-5D-5L at baseline and at the 6 month follow up to enable to computation of EQ-5D-5L scores using crosswalk mapping approach (van Hout et al., 2012) as recommended by the NICE statement at the time (NICE, 2019). QALYs will then be calculated for the trial period using the area under the curve based on EQ-5D-5L score at each time point (Richardson & Manca 2004).

DocuSign Envelope ID: 77E6F4CA-8F41-42B4-8991-9A76FB043639

5.11.5.1 Health Economics Analysis Plan

A full Health Economics analysis plan will be reviewed and signed off by the independent DMC and TSC prior to analysis.

The health economics analysis will consist of a within trial analysis and a longer term analysis using a model-based projection. The primary economic analysis will be the within-trial analysis using total costs over the trial period and smoking abstinence at 6 months follow-up. As the effects of smoking cessation on health and health care utilisation are likely to occur beyond the trial period, in order to demonstrate the effects beyond the time horizon of the trial we will undertake model-based lifetime projections to estimate the long-term cost-effectiveness of the intervention. The results will be presented as an incremental cost-effectiveness ratio (ICER) if the intervention is both more costly and more effective, with a plotted cost-effectiveness plane to visualise the uncertainties.

We will present the intervention costs in a disaggregated format, including training of smoking cessation advisors. Combining these and the operational scale within the trial, such as number of advisors trained, training organising schedule, staff capacity and so on, it will serve to provide reference for service planning or implementation. Other costs and EQ-5D-5L scores will be summarised by trial arm and time point. Unless specified in the statistical analysis, missing data will be handled with multiple imputation methods (Faria et al. 2014). The model and level of imputation for each variable will be decided based on the data distribution and missing data pattern.

5.11.5.2 Within-trial analysis

All analyses will be performed in STATA of the available version at the time of the analysis. The main analysis of the within-trial analysis will be based on the multiple imputed dataset. We will first estimate an intervention/usual care costs per quitter in each arm to compare costs and benefits of the e-cigarette intervention with usual care at the primary endpoint of the trial. We will then use appropriate regression methods to estimate incremental measures between arms for total costs (intervention costs plus general health care costs) and quit rates/QALYs. The model of regression will be decided based on the fitness of the data. Costs and outcomes will be combined to estimate the incremental cost-effectiveness ratios in terms of cost per quitter and cost per QALY, if applicable. We will assess the uncertainty surrounding the point estimates of mean incremental costs and mean incremental effectiveness by non-parametric bootstrapping methods. The estimates generated by bootstrap will be used to construct 95% confidence intervals for the incremental measures and cost-effectiveness plane to examine the uncertainties of costs and effectiveness together.

We will also undertake sensitivity analyses to assess the impact of the multiple imputation assumptions. The planned sensitivity analyses include: a complete case analysis (following the approach as in the main analysis), and alternative analyses based on Missing Not At Random assumptions.

5.11.5.3 Model-based analysis

To reflect the full impact of smoking cessation on health, we will use a model developed by one of the co-applicants at the University of York (Wu et al., 2014) to estimate lifetime ICER. This analysis will be performed in Microsoft Excel as the model was programmed in the software. The model allows participants' smoking status, costs and QALYs during the trial period to be entered as starting point and will take into account the participants' age, gender and time-based likelihood of future

Supplemental material

BMJ Publishing Group Limited (BMJ) disclaims all liability and responsibility arising from any reliance placed on this supplemental material which has been supplied by the author(s)

DocuSign Envelope ID: 77E6F4CA-8F41-42B4-8991-9A76FB043639

relapse. It attaches health utilities based on smoking status, age group and gender to project QALY gains beyond the time horizon of the trial. It also considers smoking-attributable secondary care costs and smoking-attributable mortality to project future smoking-related costs. The incremental cost per additional QALY estimate will be compared to maximum acceptable ICERs (NICE, 2013) to establish whether the intervention offers good value for money in the use of health care resources. The uncertainty surrounding the estimated lifetime ICER will be examined by probability sensitivity analysis and presented in a cost-effectiveness plane and cost-effectiveness acceptability curves CEACs (Fenwick et al., 2001). Further details on the model-based analysis will be provided in a Health Economics Analysis Plan which will be reviewed and approved by the DMC and made available prior to analysis.

5.11.6 Analysis of Qualitative Information

All interviews will be audio-recorded, and independently transcribed. After transcription the audio data will be destroyed, and data anonymised. Thematic analysis (Braun & Clarke 2006) of interview data will be considered alongside observational data and quantitative process measures such as recruitment and consent rates, retention and drop out, to provide an explanatory context for eventual trial outcomes. Thematic analysis (facilitated by QSR NVivo) will use a combination of inductive and deductive approaches to assess process elements of the trial. Initially all data will be analysed deductively, guided by the MRC guidance for complex interventions (Craig et al, 2008; Moore et al, 2015). Data will then be analysed more inductively and more broadly. This will include critiquing the conceptual approach of CoSTED, understanding any unintended consequences and reflections on the intervention from the participant perspective.

5.11.7 Analysis of Observational Information

Observation notes from each site will be coded thematically using QSR NVivo and triangulated with interview data.

5.12 Data Monitoring

5.12.1 Data Monitoring Committee (DMC)

An independent Data Monitoring committee will be convened where DMC members will be appointed and meet at regular intervals. Further details of the roles and responsibilities of the Data Monitoring Committee (DMC), including membership, relationships with other committees, decision making processes, and the timing and frequency of interim analyses (and description of stopping rules and/or guidelines where applicable) are described in detail in the CoSTED DMC Terms of Reference (ToR).

5.12.2 Interim Analyses

There is no planned interim analysis in this trial. The DMC will review recruitment, completion of data collecting and monitor the safety of participants and the conduct of the trial.

5.12.3 Data Monitoring for Harm

5.12.3.1 Adverse Event/Reaction Reporting

The principles of ICH GCP require that investigators and sponsors follow specific procedures when notifying and reporting adverse events or adverse reactions in clinical trials. These procedures are described below.

DocuSign Envelope ID: 77E6F4CA-8F41-42B4-8991-9A76FB043639

The intervention in this trial includes an e-cigarette starter kit. This is a consumer product, available over the counter and therefore the risk of adverse events is very small. The following information concerns definitions and reporting of adverse events that may arise due to the study intervention.

5.12.3.2 Procedure for those with potential contraindications

Those with health conditions and/or taking medications listed on the e-cigarette leaflet as being relative contraindications of use of the product, will be advised by their smoking cessation advisor to speak to their GP, nurse or pharmacist if they have any concerns about using an e-cigarette. As detailed in the protocol above, there is extensive evidence that e-cigarettes are much less harmful to health than smoking tobacco and therefore should only be contraindicated if there is an increase in risk relative to tobacco smoking, which is unlikely if the use is for the purpose of smoking cessation.

5.12.3.3 Identifying Adverse Events

Table 3: Adverse Event Definitions

**Adverse Event (AE)**	Any untoward medical occurrence in a patient or clinical trial participant which does not necessarily have a causal relationship with the study intervention.
**Adverse Reaction (AR)**	Any untoward and unintended response to the study intervention.
**Unexpected adverse reaction (UAR)**	An adverse reaction, the nature or severity of which is not consistent with the study intervention.
**Serious Adverse Event (SAE) or Serious Adverse Reaction (SAR)**	Any AE or AR that at any dose:<br>• results in death<br>• is life threatening<br>• requires hospitalisation or prolongs existing hospitalisation<br>• results in persistent or significant disability or incapacity<br>• is a congenital anomaly or birth defect<br>• or is another important medical condition

We will collect Serious Adverse Events and Reactions at the 1 month, 3 month and 6 month follow-ups. At 1 and 3 months participants will be asked to respond 'yes' to a text if they have been admitted to hospital since they entered into the study. Those responding will be followed-up by a researcher to identify whether the hospitalisation is a potential SAE or SAR.

Potential adverse events to the e-cigarette use will be captured in the 6-month follow up questionnaire through a list of symptoms (e.g. incidence of a dry mouth and/or throat and a dry cough) that have been known to occur following the use of an e-cigarette and measured on a Likert scale. We will also ask about any hospitalisations, which will be followed up by a researcher to identify any potential SAEs or SARs.

DocuSign Envelope ID: 77E6F4CA-8F41-42B4-8991-9A76FB043639

5.12.3.4 Investigator responsibilities relating to safety reporting

SAEs and SARs should be notified to NCTU immediately the investigator becomes aware of the event (in no circumstance should this notification take longer than 24 hours).

5.12.3.4.1 Seriousness assessment

When a participant reports an admission to hospital at any of the follow ups, the researcher must first identify whether this event has already been reported. Following this they must assess whether or not the event is serious using the definition given in **Table 1**. If the event is classified as 'serious' and there is also a possibility that the event is related to the study intervention, then an SAE form must be completed and NCTU (or delegated body) notified immediately.

5.12.3.4.2 Causality

The investigator must assess the causality of all serious events or reactions in relation to the trial therapy using the definitions in Table 4 below.

Table 4: Causality of events and reactions

Relationship	Description	Event type
Unrelated	There is no evidence of any causal relationship	Unrelated SAE
Unlikely to be related	There is little evidence to suggest that there is a causal relationship (e.g. the event did not occur within a reasonable time after administration of the trial medication). There is another reasonable explanation for the event (e.g. the participant's clinical condition or other concomitant treatment)	Unrelated SAE
Possibly related	There is some evidence to suggest a causal relationship (e.g. because the event occurs within a reasonable time after administration of the trial medication). However, the influence of other factors may have contributed to the event (e.g. the participant's clinical condition or other concomitant treatment)	SAR
Probably related	There is evidence to suggest a causal relationship and the influence of other factors is unlikely	SAR
Definitely related	There is clear evidence to suggest a causal relationship and other possible contributing factors can be ruled out.	SAR

If an SAE is considered to be related to the trial treatment, the participant will be advised to cease use of the product and to see their GP, nurse or pharmacist for an alternative treatment or product.

5.12.3.4.3 Expectedness

If there is at least a possible involvement of the trial intervention, the CI must assess the expectedness of the event. If a SAR is assessed as being unexpected it becomes a SUSAR (suspected,

DocuSign Envelope ID: 77E6F4CA-8F41-42B4-8991-9A76FB043639

unexpected, serious adverse reaction) then REC reporting guidelines apply (see Notifications sections below).

5.12.3.5 Notifications

5.12.3.5.1 Notifications by the Investigator to NCTU

If an AE meets the definition of a related SAE, a site SAE form will be completed. The following information will be recorded: description, date of onset and end date and action taken. Follow-up information should be provided as necessary. NCTU must be notified of all SAEs within 72 hours of the investigator becoming aware of the event.

Investigators should notify NCTU of any SAEs and other Notifiable Adverse Events (NAEs) occurring. The related SAE form must be completed by the trial manager with attention paid to the grading, causality and expectedness of the event.

Participants must be followed up until clinical recovery is complete. Follow-up should continue after completion of protocol treatment and/or trial follow-up if necessary. Follow-up SAE forms (clearly marked as follow-up) should be completed and emailed to NCTU as further information becomes available. Additional information and/or copies of test results etc may be provided separately. The participant must be identified by trial number, date of birth and initials only. The participant's name should not be used on any correspondence and should be blacked out and replaced with trial identifiers on any test results.

5.12.3.5.2 NCTU responsibilities

The Chief Investigator will review all SAE reports received. In the event of disagreement between the causality assessment given by the local investigator and the CI, both opinions and any justifications will be provided in subsequent reports. NCTU is responsible for the reporting of SAEs that are both related and unexpected, to the REC within 15 days of the Chief Investigator becoming aware of the event. NCTU will keep investigators informed of any safety issues that arise during the course of the trial.

5.12.4 Quality Assurance and Control

5.12.4.1 Risk Assessment

The Quality Assurance (QA) and Quality Control (QC) considerations for the CoSTED trial are based on the standard NCTU Quality Management Policy that includes a formal Risk Assessment, and that acknowledges the risks associated with the conduct of the trial and proposals of how to mitigate them through appropriate QA and QC processes. Risks are defined in terms of their impact on: the rights and safety of participants; project concept including trial design, reliability of results and institutional risk; project management; and other considerations.

QA is defined as all the planned and systematic actions established to ensure the trial is performed and data generated, documented and/or recorded and reported in compliance with the principles of GCP and applicable regulatory requirements. QC is defined as the operational techniques and activities performed within the QA system to verify that the requirements for quality of the trial related activities are fulfilled.

DocuSign Envelope ID: 77E6F4CA-8F41-42B4-8991-9A76FB043639

5.12.4.2 Central Monitoring at NCTU

A Quality Management and Monitoring Plan (QMMP) will be in place that details all planned and systematic actions established to ensure that the CoSTED trial is performed and the data are generated, documented, and reported according to the principles of Good Clinical Practice (GCP) and applicable regulatory requirements.

NCTU staff will review Case Report Form (CRF) data for errors and missing key data points. Essential trial issues, events and outputs, including defined key data points, will be detailed in the CoSTED trial Data Management Plan.

5.12.4.3 On-site Monitoring

The frequency, type and intensity of routine and triggered on-site monitoring will be detailed in the CoSTED Quality Management and Monitoring Plan (QMMP). The QMMP will also detail the procedures for review and sign-off of monitoring reports. In the event of a request for a trial site inspection by any regulatory authority, NCTU must be notified as soon as possible.

5.12.4.3.1 Direct access to participant records

Participating investigators must agree to allow trial related monitoring, including audits, REC review and regulatory inspections, by providing access to source data and other trial related documentation as required. Participant consent for this must be obtained as part of the informed consent process for the trial.

5.12.4.4 Trial Oversight

Trial oversight is intended to preserve the integrity of the trial by independently verifying a variety of processes and prompting corrective action where necessary. The processes reviewed relate to participant enrolment, consent, eligibility, and allocation to trial groups; adherence to trial interventions and policies to protect participants, including reporting of harms; completeness, accuracy and timeliness of data collection; and will verify adherence to applicable policies detailed in the Compliance section of the protocol. Independent trial oversight complies with the NCTU trial oversight policy.

In multi-centre trials this oversight is considered and described both overall and for each recruiting centre by exploring the trial dataset or performing site visits as described in the CoSTED Quality Management and Monitoring Plan.

5.12.4.4.1 Core Trial Team (TT)

The Core Trial Team (TT) will be set up to assist with developing the design, co-ordination and day to day operational issues in the management of the trial, including budget management. The membership, frequency of meetings, activity (including trial conduct and data review) and authority will be covered in the TT terms of reference. Membership will include the CIs, Trial Managers, Senior Research Associates, a trial administrator and a research team member from each of the sites. Recruitment per site, number of sites opened, total number of participants and other metrics available from online screening logs will be reviewed monthly by the trial team during trial team meetings.

DocuSign Envelope ID: 77E6F4CA-8F41-42B4-8991-9A76FB043639

5.12.4.4.2 Trial Management Group (TMG)

A Trial Management Group (TMG) will be set up to assist with developing the design, co-ordination and strategic management of the trial. The membership, frequency of meetings, activity (including trial conduct and data review) and authority will be covered in the TMG terms of reference. Membership will include the CIs, PIs, Statistician, Health Economist, Trial Managers, Senior Research Associates and a trial administrator.

5.12.4.4.3 Independent Trial Steering Committee (TSC)

The Independent Trial Steering Committee (TSC) is the independent group responsible for oversight of the trial in order to safeguard the interests of trial participants. The TSC provides advice to the CI, NCTU, the funder and sponsor on all aspects of the trial through its independent Chair. The membership, frequency of meetings, activity (including trial conduct and data review) and authority will be covered in the TSC terms of reference.

5.12.4.4.4 Independent Data Monitoring Committee (IDMC)

The Independent Data Monitoring Committee (IDMC) is the only oversight body that has access to unblinded accumulating comparative data. The IDMC is responsible for safeguarding the interests of trial participants, monitoring the accumulating data and making recommendations to the TSC on whether the trial should continue as planned. The membership, frequency of meetings, activity (including review of trial conduct and data) and authority will be covered in the IDMC terms of reference. The IDMC will consider data in accordance with the statistical analysis plan and will advise the TSC through its Chair.

5.12.4.4.5 Patient and Public Involvement (PPI)

PPI representatives have been involved in the development of this work. Up to four PPI representatives will be involved in choosing the device to be used in the trial and in the development of the intervention. Two PPI representatives will be appointed to the TSC to advise on all aspects of the conduct of the trial.

5.12.4.4.6 Trial Sponsor

The NNUH is the trial sponsor. The role of the sponsor is to take on responsibility for securing the arrangements to initiate, manage and finance the trial. The Sponsor is responsible for ensuring that the study meets the relevant standards and makes sure that arrangements are put and kept in place for management, monitoring and reporting. The NNUH has delegated some Sponsor’s activities to the CI and NCTU, these are documented in the Delegation of Activities (schedule 6 of the Collaboration Agreement).

DocuSign Envelope ID: 77E6F4CA-8F41-42B4-8991-9A76FB043639

6 Ethics and Dissemination

6.1 Research Ethics and Health Research Authority Approval

Before initiation of the trial at any clinical site, the protocol, all informed consent forms and any material to be given to the prospective participant will be submitted to the relevant REC and to HRA for approval. Any subsequent amendments to these documents will be submitted for further approval.

The rights of the participant to refuse to participate in the trial without giving a reason must be respected. After the participant has entered the trial, the clinician remains free to give alternative treatment to that specified in the protocol, at any stage, if s/he feels it to be in the best interest of the participant. The reasons for doing so must be recorded. After randomisation the participant must remain within the trial for the purpose of follow up and data analysis according to the treatment option to which they have been allocated. However, the participant remains free to change their mind at any time about the protocol treatment and follow-up without giving a reason and without prejudicing their further treatment.

6.2 Competent Authority Approvals

This is not a Clinical Trial of an Investigational Medicinal Product (IMP) as defined by the EU Directive 2001/20/EC. Therefore, a CTA is not required in the UK.

6.3 Other Approvals

Documentation will need to be submitted to the R&D Department at each NHS Site in order to gain confirmation of capacity and capability prior to the study being initiated at that site. Confirmation from the site will take the form of a site agreement signed by both the Sponsor/NCTU and the relevant site.

A copy of the local R&D approval and of the Participant Information Sheet (PIS) and consent form on local headed paper must be forwarded to the co-ordinating centre before participants are randomised to the trial.

The protocol has received formal approval and methodological, statistical, clinical and operational input from the NCTU Protocol Review Committee.

6.4 Amendments

Amendments to the Protocol and other documents (e.g. changes to eligibility criteria, outcomes, sample size calculations, analyses) will be agreed by the TMG. Such amendments will be forwarded to the Sponsor for authorisation and will then be submitted to the Health Research Authority or Ethics Committee for categorisation and approval. Once the amendment has been submitted by NCTU it will be sent to relevant sites for consideration in accordance with standard HRA processes and timescales. Amendments must not be implemented until HRA approval is received and sites have either confirmed acceptance or, no objection has been received within the defined timescale. Notification will be sent by NCTU to trial personnel to confirm when an amendment can be implemented.

DocuSign Envelope ID: 77E6F4CA-8F41-42B4-8991-9A76FB043639

6.5 Consent

Patients will be provided with a Patient Information Sheet (PIS) and given time to read it fully. Following a discussion with a medical qualified investigator or suitable trained and authorised delegate, any questions will be satisfactorily answered and if the participant is willing to participate, written informed consent will be obtained. During the consent process it will be made completely and unambiguously clear that the participant is free to refuse to participate in all or any aspect of the trial, at any time and for any reason, without incurring any penalty or affecting their treatment.

Consent will be re-sought if new information becomes available that affects the participant's consent in any way. This will be documented in a revision to the patient information sheet and the participant will be asked to sign an updated consent form. These will be approved by the ethics committee prior to their use.

A copy of the approved consent form is available from the NCTU trial team.

6.5.1 Consent in Ancillary Studies

Consent for involvement in wider qualitative studies or trial sub-studies will be sought and approval gained at a later date if this becomes appropriate and desirable.

6.6 Confidentiality

Any paper copies of personal trial data will be kept at the participating site in a secure location with restricted access. Following consent, identifiable data will be kept on the trial database to allow authorised members of the trial team to contact patients in order to arrange appointments/assessments. Only authorised trial team members will have password access to this part of the database. This information will be securely destroyed 5 years after the end of the trial.

Confidentiality of patients' personal data is ensured by not collecting patient names on CRFs and limiting access to personal information held on the database at NCTU. At trial enrolment the patient will be issued a participant identification number, and this will be the primary identifier for the patient, with secondary identifiers of month and year of birth and initials.

The patient's consent form will carry their name and signature. These will be kept at the trial site, with a copy sent to NCTU for monitoring purposes. This copy will be destroyed once checks are complete. Consent forms will not be kept with any additional patient data.

6.7 Declaration of Interests

The investigators named on the protocol have no financial or other competing interests that impact on their responsibilities towards the scientific value or potential publishing activities associated with the trial.

6.8 Indemnity

NHS holds insurance to cover participants for injury caused by their participation in the clinical trial.

6.9 Finance

CoSTED is funded by the National Institute for Health Research (NIHR) [Health Technology Assessment (HTA) Programme (Reference: NIHR129438)]. The views expressed are those of the

DocuSign Envelope ID: 77E6F4CA-8F41-42B4-8991-9A76FB043639

author(s) and not necessarily those of the NIHR or the Department of Health and Social Care. It is not expected that any further external funding will be sought.

6.10 Archiving

The investigators agree to archive and/or arrange for secure storage of CoSTED trial materials and records for 5 years after the close of the trial unless otherwise advised by the NCTU

6.11 Access to Data

Requests for access to trial data will be considered, and approved in writing where appropriate, after formal application to the TMG/TSC. Considerations for approving access are documented in the TMG/TSC Terms of Reference.

6.12 Ancillary and Post-trial Care

Following trial completion, all participants will be signposted to usual care support for smoking cessation and advised to seek smoking cessation support from their GPs.

6.13 Publication Policy

6.13.1 Trial Results

The results of the trial will be disseminated regardless of the direction of effect. Ownership of the data arising from the study resides with the trial team. The publication policy will be in line with rules of the International Committee of Medical Journal Editors. The TMG will agree a publication plan and decide on authorship, with any difficulties being resolved by the TSC. Outputs from the trial will be available on the trial website for participants to access, with the website address detailed in the PIS to enable access.

6.13.2 Dissemination

On commencement of the trial we will agree a dissemination plan according to NIHR guidance. We will liaise with the NIHR dissemination centre to agree the plan for maximum impact of trial outcomes. The overall aim of dissemination is to raise expert and general public awareness of the intervention and the planned programme of implementation, if proven effective. We will directly inform commissioners of smoking cessation services and managers and ED department leads. One of our main mechanisms for UK dissemination will be via partnership working with ASH (Action on Smoking and Health), Public Health England, and the Royal College of Emergency Medicine. Outputs of the trial will be:

1. Academic outputs: publications in leading open access journals widely accessible to international audiences: trial protocol (open access trials registry), main outcome paper (BMJ/Lancet), health economics paper (Journal of Health Economics), process evaluation paper (Addiction/Nicotine and Tobacco research); and presentations/abstracts at academic conferences (reaching smoking cessation, Emergency medicine health professionals and public health audiences). During the dissemination period the results will be presented at the International Conference on Emergency Medicine and the International Health Congress, Oxford.

DocuSign Envelope ID: 77E6F4CA-8F41-42B4-8991-9A76FB043639

2. Recommendations for service commissioners and managers: study findings and recommendations for future ED practice will be disseminated via a one day national dissemination event. Brief reports will be prepared for commissioners and Public Health England in the form of an 'evidence briefing', succinctly summarising key findings. Presentations will be held in each study area, and beyond through working with our networks of contacts, to disseminate findings face to face to service managers and healthcare professionals, including ED staff and stop smoking services. We will align with established groups for presentations of research findings, such as the regional clinical research networks.

3. Dissemination to the public: a social media campaign will ensure wide dissemination to the general public, using Twitter and Facebook. We will write press releases and produce a podcast summarising study findings in a public friendly format.

Throughout the project, a project website will be maintained to feed-back to the public key milestones and to inform the wider population of project progress. This will be linked to recruiting hospitals websites. Through the UEA Addictions Research Group, regular twitter posts will keep the wider public informed and engaged. On completion of the project, we will work with our PPI members to ensure adequate and appropriate public dissemination. We will engage with health professionals through presentations at clinical research network events in each recruitment hub area.

Results will be presented at the Royal College of Emergency Medicine Annual Scientific Conference, Public Health England Annual Conference and the Society for Research on Nicotine and Tobacco annual conference. There will be a national dissemination event with members of the trial team, staff from primary and secondary care, stop smoking and public health services, and members of the public.

We will also produce a public facing summary of the research findings working in conjunction with ASH and the National Centre for Smoking Cessation and Training, whom our research team have established collaborative links with.

6.13.3 Authorship

Authorship will be agreed upfront through discussion and agreement at the TMG of a publication plan. The TMG will nominate a writing group, which will consist of members of the TMG and will be responsible for drafting the manuscript(s) for publication. These individuals will be named on the final publication.

6.13.4 Reproducible Research

The full protocol, statistical analysis plan, qualitative and health economics analysis plans, and anonymised datasets will be published in an online open access repository.

7 Ancillary Studies

No ancillary studies are currently planned. Any that are proposed during the lifetime of the trial will require funding applications to be made and will be submitted for ethical approval prior to initiation.

DocuSign Envelope ID: 77E6F4CA-8F41-42B4-8991-9A76FB043639

8 Protocol Amendments

Document Name	Version No.	Effective Date	Reason for Change

DocuSign Envelope ID: 77E6F4CA-8F41-42B4-8991-9A76FB043639

9 References

Addiction Ontology. (2021).E-cigarette. Qeios. doi:10.32388/0WX80U.

ASH. Progress towards smokefree mental health services: Findings from a survey of mental health trusts in England, 2019a. Available from: https://ash.org.uk/wp-content/uploads/2019/10/PHE-mental-health-trust-2019-survey-full-report_v12.pdf

ASH. Use of e-cigarettes among adults in Great Britain. 2019b [cited 2019 Dec 7]. Available from: https://ash.org.uk/wp-content/uploads/2019/09/Use-of-e-cigarettes-among-adults-2019.pdf

Beaglehole R, Bates C, Youdan B, Bonita R. Nicotine without smoke: fighting the tobacco epidemic with harm reduction. The Lancet. 2019 Aug 31;394(10200):718–20.

Bellg AJ, Borrelli B, Resnick B, Hecht J, Minicucci DS, Ory M, et al. Enhancing treatment fidelity in health behavior change studies: best practices and recommendations from the NIH Behavior Change Consortium. Health Psychol Off J Div Health Psychol Am Psychol Assoc. 2004 Sep;23(5):443–51.

Bensberg M, Kennedy M. A framework for health promoting emergency departments. Health Promot Int. 2002 Jun 1;17(2):179–88.

Bernstein SL, D'Onofrio G, Rosner J, O'Malley S, Makuch R, Busch S, et al. Successful Tobacco Dependence Treatment in Low-Income Emergency Department Patients: A Randomized Trial. Ann Emerg Med. 2015 Aug 1;66(2):140–7.

Blount BC, Karwowski MP, Shields PG, Morel-Espinosa M, Valentin-Blasini L, Gardner M, et al. Vitamin E Acetate in Bronchoalveolar-Lavage Fluid Associated with EVALI. N Engl J Med. 2019 Dec 20; NEJMoa1916433.

Boudreaux ED, Baumann BM, Friedman K, Ziedonis DM. (2005) Smoking Stage of Change and Interest in an Emergency Department-based Intervention. *Acad Emerg Med*. Mar;12(3):211–8.

Braun V, Clarke V. Using thematic analysis in psychology. *Qual Res Psychol*. 2006 Jan;3(2):77–101

Bullen C, Howe C, Laugesen M, McRobbie H, Parag V, Williman J, et al. Electronic cigarettes for smoking cessation: a randomised controlled trial. The Lancet. 2013 Nov 16;382(9905):1629–37.

Chan SSC, Leung DYP, Abdullah ASM, Wong VT, Hedley AJ, Lam T-H. (2011) A randomized controlled trial of a smoking reduction plus nicotine replacement therapy intervention for smokers not willing to quit smoking. *Addict Abingdon Engl*. Jun;106(6):1155–63.

Chan AW, Tetzlaff JM, Altman DG et al. SPIRIT (2013a). Statement: Defining Protocol Items for Clinical Trials. *Ann Intern Med*; 158:200-207.

Chan AW, Tetzlaff, Gotzsche et al. SPIRIT (2013b) explanation and elaboration: guidance for protocols of clinical trials. *BMJ* 2013; 346: e7586.

Cheung KW, Wong IW, Fingrut W, Tsai APY, Ke SR, Shojaie S, et al. Randomized controlled trial of emergency department initiated smoking cessation counselling and referral to a community counselling service. Can J Emerg Med. 2018 Jul;20(4):556–64.

Craig P, Dieppe P, Macintyre S, et al. Developing and evaluating complex interventions: the new Medical Research Council guidance. BMJ2008;337:a1655.

Dawkins L, Ford A, Bauld L, Balaban S, Tyler A, Cox S. A cross sectional survey of smoking characteristics and quitting behaviour from a sample of homeless adults in Great Britain. Addict Behav. 2019 Aug 1;95:35–40.

DocuSign Envelope ID: 77E6F4CA-8F41-42B4-8991-9A76FB043639

Department of Health. Towards a Smokefree Generation – A tobacco control plan for England. DOH 2017.

Drummond C, Deluca P, Coulton S, Bland M, Cassidy P, Crawford M, et al. (2014) The effectiveness of alcohol screening and brief intervention in emergency departments: a multicentre pragmatic cluster randomized controlled trial. PloS One. 9(6):e99463.

EuroQol Research Foundation. EQ-5D-5L User Guide: Basic information on how to use the EQ-5D-5L instrument (version 3.0)2019 17/06/2020. Available at: https://euroqol.org/publications/user-guides/.

English TM, Smith TB, Song X, Whitman MV. Barriers to electronic cigarette use. Public Health Nurs Boston Mass. 2018;35(5):363–8.

Faria R, Gomes M, Epstein D, et al. A guide to handling missing data in cost-effectiveness analysis conducted within randomised controlled trials. Pharmacoeconomics. 2014;32(12):1157-70. https://www.ncbi.nlm.nih.gov/pubmed/25069632

Fenwick E, Claxton K, Sculpher M. Representing uncertainty: the role of cost-effectiveness acceptability curves. Health Econ. 2001;10(8):779–87.

George J, Hussain M, Vadiveloo T, Ireland S, Hopkinson P, Struthers AD, et al. Cardiovascular Effects of Switching From Tobacco Cigarettes to Electronic Cigarettes. J Am Coll Cardiol [Internet]. 2019 Nov 15 [cited 2019 Dec 7]; Available from: http://www.onlinejacc.org/content/early/2019/11/12/j.jacc.2019.09.067

Hajek P, Phillips-Waller A, Przulj D, Pesola F, Myers Smith K, Bisal N, et al. A Randomized Trial of E-Cigarettes versus Nicotine-Replacement Therapy. N Engl J Med. 2019 Jan 30;380(7):629–37.

Halpern SD, Harhay MO, Saulsgiver K, Brophy C, Troxel AB, Volpp KG. A Pragmatic Trial of ECigarettes, Incentives, and Drugs for Smoking Cessation. *N Engl J Med*. 2018 May 23;378(24):2302–10.

Hartmann-Boyce J, McRobbie H, Lindson N, Bullen C, Begh R, Theodoulou A, Notley C, Rigotti NA, Turner T, Butler AR, Fanshawe TR, Hajek P. Electronic cigarettes for smoking cessation. Cochrane Database of Systematic Reviews 2021, Issue 4. Art. No.: CD010216.

Heatherton T, Kozlowski LT, Frecker RC, Fagerstrom K. The Fagerstrom test for nicotine dependence: a revision of the Fagerstrom tolerance questionnaire. British Journal of Addiction. 1991; 86: 1119-1127.

Hennrikus D, Joseph AM, Lando HA, Duval S, Ukestad L, Kodl M & Hirsch AT. Effectiveness of a Smoking Cessation Program for Peripheral Artery Disease Patients: A Randomized Controlled Trial. J *Am Coll Cardiol*. 2010 Dec, 56 (25) 2105–2112.

Keene J, Rodriguez J. (2007) Are mental health problems associated with use of Accident and Emergency and health-related harm? *Eur J Public Health*. Mar 28;17(4):387–93.

Kotz D, Brown J, West R. Predictive validity of the Motivation To Stop Scale (MTSS): A single-item measure of motivation to stop smoking. Drug and Alcohol Dependence. 2013, 128: 15-19.

McFall M, Saxon AJ, Malte CA, Chow B, Bailey S, Baker DG, et al. Integrating Tobacco Cessation Into Mental Health Care for Posttraumatic Stress Disorder: A Randomized Controlled Trial. JAMA. 2010 Dec 8;304(22):2485–93.

DocuSign Envelope ID: 77E6F4CA-8F41-42B4-8991-9A76FB043639

Lemhoefer C, Rabe GL, Wellmann J, Bernstein SL, Cheung KW, McCarthy WJ, et al. (2017) Emergency Department–Initiated Tobacco Control: Update of a Systematic Review and Meta-Analysis of Randomized Controlled Trials. *Prev Chronic Dis*; 14: 160434. http://dx.doi.org/10.5888/pcd14.160434

McNeill A, Brose LS, Calder R, Bauld L & Robson D (2018). Evidence review of e-cigarettes and heated tobacco products 2018. A report commissioned by Public Health England. London: Public Health England. Available from: https://assets.publishing.service.gov.uk/government/uploads/system/uploads/attachment_data/file/684963/Evidence_review_of_e-cigarettes_and_heated_tobacco_products_2018.pdf

McNeill, A., Brose, L.S., Calder, R., Simonavicius, E. and Robson, D. (2021). Vaping in England: An evidence update including vaping for smoking cessation, February 2021: a report commissioned by PHE. London: PHE. https://assets.publishing.service.gov.uk/government/uploads/system/uploads/attachment_data/file/962221/Vaping_in_England_evidence_update_February_2021.pdf

Moore G, Audrey S, Barker M, Bonell C, Hardeman W, Moore L, et al. Process evaluation of complex interventions: Medical Research Council guidance. BMJ 2015; 350:h1258

National Centre for Smoking Cessation and Training. Stopping Smoking in Pregnancy: A briefing for maternity care providers. 2019. https://www.ncsct.co.uk/publication_briefing_for_midwifery_staff.php

NHS. *The NHS Long Term Plan*. (2019) Available from: https://www.longtermplan.nhs.uk/online-version/chapter-2-more-nhs-action-on-prevention-and-health-inequalities/smoking/. [Accessed April 28 2019].

NHS Improvement. Reference costs [Internet]. [cited 2019 Dec 7]. Available from: https://improvement.nhs.uk/resources/reference-costs/

NHS. Stopping smoking in pregnancy: Your pregnancy and baby guide. 2019a. https://www.nhs.uk/conditions/pregnancy-and-baby/smoking-pregnant/ (accessed 28 August 2019)

NICE. Guide to the methods of technology appraisal 2013. Available from: https://www.nice.org.uk/process/pmg9/chapter/foreword.

NICE. Position statement on use of the EQ-5D-5L value set for England (updated October 2019) 2019 [updated Oct 2019; cited 2019 4th November]. https://www.nice.org.uk/about/what-we-do/our-programmes/nice-guidance/technology-appraisal-guidance/eq-5d-5l

Nilsen P, Baird J, Mello MJ, Nirenberg T, Woolard R, Bendtsen P, et al. A systematic review of emergency care brief alcohol interventions for injury patients. J Subst Abuse Treat. 2008 Sep 1;35(2):184–201.

Notley C, Ward E, Dawkins L, Holland R. The unique contribution of e-cigarettes for tobacco harm reduction in supporting smoking relapse prevention. *Harm Reduct J*. 2018 20;15(1):31.

Notley C, Gentry S, Livingstone-Banks J, Bauld L, Perera R, Hartmann-Boyce J. Incentives for smoking cessation. Cochrane Database of Systematic Reviews 2019, Issue 7. Art. No.: CD004307. DOI: 10.1002/14651858.CD004307.pub6. Accessed 02 February 2021.

Office for National Statistics. (2018a) *Likelihood of smoking four times higher in England's most deprived areas than least deprived*. Available from:

DocuSign Envelope ID: 77E6F4CA-8F41-42B4-8991-9A76FB043639

https://www.ons.gov.uk/peoplepopulationandcommunity/healthandsocialcare/drugusealcoholandsmoking/articles/likelihoodofsmokingfourtimeshigherinenglandsmostdeprivedareasthanleastdeprived/2018-03-14 [Accessed 28 April 2019].

Office for National Statistics. (2018b) Adult smoking habits in the UK. Available from: https://www.ons.gov.uk/releases/adultsmokinghabitsintheuk2018 [Accessed 28 April 2019].

Pieterse ME, Seydel ER, DeVries H, Mudde AN, Kok GJ. Effectiveness of a Minimal Contact Smoking Cessation Program for Dutch General Practitioners: A Randomized Controlled Trial. Prev Med. 2001 Feb 1;32(2):182–90.

PSSRU. Unit Costs of Health and Social Care 2018. [cited 2019 Dec 7]. Available from: https://www.pssru.ac.uk/project-pages/unit-costs/unit-costs-2018/

Public Health England. (2017) Cost of smoking to the NHS in England: 2015. Available from: https://www.gov.uk/government/publications/cost-of-smoking-to-the-nhs-in-england-2015/cost-of-smoking-to-the-nhs-in-england-2015 [Accessed 28 April 2019]

Raiff BR, Faix C, Turturici M, Dallery J. Breath carbon monoxide output is affected by speed of emptying the lungs: Implications for laboratory and smoking cessation research. Nicotine Tob Res [Internet]. 2010 Aug [cited 2019 Aug 7];12(8):834–8. Available from: https://www.ncbi.nlm.nih.gov/pmc/articles/PMC2910872/

Ramsay J, Richardson J, Carter YH, Davidson LL, Feder G. Should health professionals screen women for domestic violence? Systematic review. BMJ. 2002 Aug 10;325(7359):314.

Reitsma MB, Fullman N, Ng M, Salama JS, Abajobir A, Abate KH, et al. (2017) Smoking prevalence and attributable disease burden in 195 countries and territories, 1990–2015: a systematic analysis from the Global Burden of Disease Study 2015. The Lancet. May 13;389(10082):1885–906.

Richardson G, Manca A. Calculation of quality adjusted life years in the published literature: a review of methodology and transparency. Health Econ. 2004 Dec;13(12):1203–10.

Robson D, Cole F, Jalasi S, Boojharut B, Smith S, Thompson S, et al. Smoking cessation and serious mental illness: a service evaluation of a drop-in stop smoking clinic on an acute inpatient unit. J Clin Nurs. 2013;22(3–4):405–13.

Royal College of Midwives. Position statement: Support to quit smoking in pregnancy. 2019. https://www.rcm.org.uk/media/3394/support-to-quit-smoking-in-pregnancy.pdf

Royal College of Physicians, Royal College of Psychiatrists. (2013) *Smoking and mental health*. London: RCP. Royal College of Psychiatrists Council Report CR178RCP. Available from: https://www.rcplondon.ac.uk/projects/outputs/smoking-and-mental-health [Accessed 28 April 2019].

Royal College of Physicians. Nicotine without smoke: Tobacco harm reduction [Internet]. RCP London. 2016. Available from: https://www.rcplondon.ac.uk/projects/outputs/nicotine-without-smoketobacco-harm-reduction

Royal College of Physicians (2018) *Hiding in plain sight: Treating tobacco dependency in the NHS*. London: RCP. Available from: https://www.rcplondon.ac.uk/projects/outputs/hiding-plain-sighttreating-tobacco-dependency-nhs. [Accessed 28 April 2019].

Rudge GM, Mohammed MA, Fillingham SC, Girling A, Sidhu K, Stevens AJ. (2013) The Combined Influence of Distance and Neighbourhood Deprivation on Emergency Department Attendance in a

DocuSign Envelope ID: 77E6F4CA-8F41-42B4-8991-9A76FB043639

Large English Population: A Retrospective Database Study. Jiang B, editor. PLoS ONE. Jul 16;8(7):e67943.

Shahab L, Goniewicz ML, Blount BC, Brown J, McNeill A, Alwis KU, et al. Nicotine, Carcinogen, and Toxin Exposure in Long-Term E-Cigarette and Nicotine Replacement Therapy Users: A Crosssectional Study. Ann Intern Med. 2017 Mar 21;166(6):390–400.

Shields W, McDonald E, McKenzie L, Wang M-C, Walker AR, Gielen A. Utilizing the pediatric emergency department to deliver tailored safety messages: Results of a randomized controlled trial. Pediatr Emerg Care. 2013 May;29(5):628–34.

Smoking In England 2021 | Welcome [Internet]. Available from: http://www.smokinginengland.info/ [Accessed 19th May 2021]

SRNT Subcommittee on Biochemical Verification. Biochemical verification of tobacco use and cessation. Nicotine Tob Res. 2002 May;4(2):149-59. doi: 10.1080/14622200210123581. PMID: 12028847.

Stanley B, Brown GK. Safety Planning Intervention: A Brief Intervention to Mitigate Suicide Risk. Cogn Behav Pract. 2012 May 1;19(2):256–64.

van Hout B, Janssen MF, Feng Y-S, Kohlmann T, Busschbach J, Golicki D, et al. Interim scoring for the EQ-5D-5L: mapping the EQ-5D-5L to EQ-5D-3L value sets. Value Health J Int Soc Pharmacoeconomics Outcomes Res. 2012 Aug;15(5):708–15.

Walker N, Parag V, Verbiest M, Laking G, Laugesen M, Bullen C. Nicotine patches used in combination with e-cigarettes (with and without nicotine) for smoking cessation: a pragmatic, randomised trial. Lancet. Respiratory Medicine 2020;8(1):54-64.

West R, Hajek P, Stead L, Stapleton J. Outcome criteria in smoking cessation trials: proposal for a common standard. Addiction. 2005;100(3):299–303.

Wu Q, Parrott S, Godfrey C, Gilbert H, Nazareth I, Leurent B, et al. Cost-effectiveness of computer-tailored smoking cessation advice in primary care: a randomized trial (ESCAPE). Nicotine Tob Res Off J Soc Res Nicotine Tob. 2014 Mar;16(3):270–8.
