# Supplementary material for: Cessation of Smoking Trial in the Emergency Department (COSTED): a multicentre randomised controlled trial
Source: Emerg Med J. 2024 Mar 26;41(5):276–82. doi: 10.1136/emermed-2023-213824 (PMC11041600; doi:10.1136/emermed-2023-213824)
Supplement: Supplementary data [file emermed-2023-213824supp002.pdf]

Supplementary material- Sensitivity analysis for missing= smoking assumption

Sensitivity analyses were conducted using the pattern mixture methods of [21] to allow for missing not at random. This method uses the odds ratio rather than the relative risk as presented in the main text. The results were assessed under varying assumptions of how much greater odds those who dropped out were to smoke compared to those who did not drop-out. This ranged from assuming that the drop-outs always smoked (log-delta = 0) to assuming that the drop-outs were equally likely to smoke (log-delta=1). The results are given in the Table below and show that provided the drop-outs have less than 0.2 times the odds of being abstinent than those who remain the intervention is statistically significant. Even under the assumption that the drop-outs are equally likely to smoke as those who remain the estimated odds ratio is still larger than 1.5, but no longer statistically significant.

Table 1: Estimates of the odds ratio of abstinence in the intervention group compared to control under different missing data assumptions.

**Log-odds of drop-outs smoking compared to non-dropouts (Delta)**	**OR (95% CI) (unadjusted)**	**OR (95% CI) (adjusted)**
0	1.82 (1.04,3.21)	1.82 (1.04,3.21)
0.1	1.80 (1.02,3.17)	1.78 (1.01,3.14)
0.2	1.78 (1.01,3.14)	1.75 (0.99,3.08)
0.3	1.75 (0.99,3.10)	1.71 (0.97,3.03)
0.4	1.74 (0.98,3.07)	1.69 (0.95,2.98)
0.5	1.72 (0.97,3.04)	1.66 (0.94,2.94)
0.6	1.70 (0.96,3.01)	1.64 (0.93,2.90)
0.7	1.69 (0.95,2.99)	1.62 (0.91,2.86)
0.8	1.67 (0.94,2.96)	1.60 (0.90,2.82)
0.9	1.66 (0.93,2.94)	1.58 (0.89,2.79)
1	1.64 (0.93,2.92)	1.56 (0.88,2.76)
